# Piezo1–Src signaling rapidly disrupts brain endothelial barrier integrity

**DOI:** 10.1186/s12987-026-00859-6

**Published:** 2026-08-13

**Authors:** Yu Iida, Taisuke Akimoto, Kagemichi Nagao, Akane Nagasako, Michiko Endo, Yayoi Kimura, Makoto Ohtake, Tetsuya Yamamoto, Yoshihiro Ishikawa, Masanari Umemura

**Affiliations:** 1 Department of Neurosurgery, Yokohama City University Graduate School of Medicine, Yokohama, Kanagawa Japan; 2 Department of Neurosurgery, Yokohama Municipal Minato Red Cross Hospital, Yokohama, Japan; 3 Department of Molecular Physiology, Jikei University Graduate School of Medicine, 3-25-8, Nishishimbashi, Minato-ku, Tokyo 105-8461 Japan; 4 Advanced Medical Research Center, Yokohama City University, Yokohama, Kanagawa Japan; 5 Yokohama City University, Yokohama, Kanagawa Japan

**Keywords:** Piezo1, Src, Blood–brain barrier, Tight junction, Phosphoproteomics, Human brain microvascular endothelial cells, Ischemia–reperfusion

## Abstract

**Background:**

Blood-brain barrier integrity is essential for central nervous system homeostasis, yet the mechanisms underlying ultra-acute barrier dysfunction after abrupt hemodynamic changes remain incompletely understood. Piezo1 is a mechanosensitive Ca²⁺ channel expressed in endothelial cells and activated by mechanical stimuli such as shear stress, but its contribution to rapid brain endothelial barrier disruption remains unclear. We investigated whether Piezo1 activation induces an ultra-acute disruption of the endothelial barrier and explored the underlying signaling mechanisms.

**Methods:**

Human brain microvascular endothelial cells (HBEC-5i) were stimulated with the Piezo1 agonist Yoda1 in the presence or absence of the Src inhibitor saracatinib, followed by barrier-function assessment and phosphoproteomic profiling. To assess in vivo relevance, vascular permeability was evaluated within minutes after reperfusion in a mouse cerebral ischemia–reperfusion model treated with the mechanosensitive channel inhibitor GsMTx4.

**Results:**

Comprehensive phosphoproteomic profiling after Piezo1 activation revealed enrichment of phosphorylation events associated with cell junctions, adhesion, and cytoskeletal organization. Yoda1 induced rapid phosphorylation of junction-associated proteins, including occludin at Tyr287, and caused acute barrier disruption in HBEC-5i cells. Saracatinib attenuated occludin Tyr287 phosphorylation and preserved barrier integrity, supporting the involvement of Src-family kinase-sensitive signaling in this response. In mice, GsMTx4 significantly attenuated vascular permeability within minutes after reperfusion, consistent with the in vitro observations and supporting a role for mechanosensitive channel-associated signaling in ultra-acute blood–brain barrier leakage immediately after flow restoration.

**Conclusions:**

This study provides the first phosphoproteomic characterization of rapid phosphorylation changes associated with Piezo1 activation in human brain endothelial cells. Guided by these phosphoproteomic signatures, our data support the involvement of saracatinib-sensitive signaling, accompanied by occludin Tyr287 phosphorylation, in rapid endothelial barrier disruption. These findings suggest that Piezo1-associated signaling may contribute to ultra-acute BBB dysfunction and provide a rational for further investigation in the context of abrupt hemodynamic stress and cerebral revascularization.

**Supplementary Information:**

The online version contains supplementary material available at https://doi.org/10.1186/s12987-026-00859-6.

## Introduction

Blood-brain barrier (BBB) integrity is essential for central nervous system homeostasis. BBB disruption after ischemia-reperfusion is frequently conceptualized as comprising an early increase in permeability followed by delayed structural injury [1]. Although BBB dysfunction after ischemia-reperfusion has been studied extensively, most prior work has focused on delayed injury mechanisms involving inflammation, leukocyte infiltration, and matrix degradation [1, 2]. By contrast, the molecular events that trigger ultra-acute barrier dysfunction within minutes after reperfusion remain incompletely understood. This early phase is clinically important because early BBB dysfunction after cerebral reperfusion is associated with subsequent hemorrhagic complications [3], yet the upstream molecular triggers remain poorly defined.

The BBB is regulated by multiple components of the neurovascular unit, including endothelial cells, pericytes, and astrocytes. However, paracellular permeability is primarily restricted by endothelial junctional complexes and is therefore a principal immediate determinant of rapid permeability changes. Brain microvascular endothelial cells maintain exceptionally low permeability through highly specialized junctional complexes while being continuously exposed to pulsatile flow and pressure fluctuations. This raises the possibility that mechanosensitive signaling participates in the endothelial response to abrupt hemodynamic stress.

Piezo1 is a mechanosensitive Ca²⁺ channel expressed in endothelial cells and activated by physical stimuli such as shear stress and membrane stretch [4, 5]. Piezo1 has been implicated in vascular development, flow sensing, and endothelial signaling [5, 6], and studies in non-brain endothelial systems have shown that Piezo1 activation can promote endothelial opening and barrier destabilization [7–9]. However, whether Piezo1-associated signaling contributes to rapid disruption of brain endothelial barrier integrity remains unclear. In particular, the phosphorylation programs engaged by Piezo1 activation in brain endothelial cells have not been comprehensively characterized.

In the present study, we combined phosphoproteomic profiling, barrier assays, and a mouse ischemia-reperfusion model to investigate signaling pathways associated with Piezo1-dependent endothelial barrier disruption. We hypothesized that Piezo1 activation engages Src-associated phosphorylation signaling that contributes to rapid tight junction dysfunction in brain endothelial cells under ultra-acute reperfusion stress.

## Methods

### Cell culture

Human brain microvascular endothelial cells (HBEC-5i; ATCC CRL-3245) were cultured in DMEM/F-12 (FUJIFILM Wako, 048-29785) containing 10% FBS and Endothelial Cell Growth Supplement (Takara Bio, C-39211) on gelatin-coated plates. Gelatin (porcine skin, type A; FUJIFILM Wako, 071-03151) was applied for ≥ 45 min at 37 °C and aspirated before seeding. Cells were maintained at 37 °C, 5% CO₂, passaged at confluence, and only passages ≤ 7 were used.

## Reagents

Defactinib (VS-6063; MedChemiExpress) and saracatinib (AZD0530; Selleck) were used as FAK/PYK2 and Src inhibitors, respectively. Yoda1 (Sigma-Aldrich, SML1558) was dissolved in DMSO to generate stock solutions. GsMTx4 peptide (Peptide Institute, 4393-s) was dissolved in sterile water. VEGF165 was from Nacalai Tesque (21957-24). Final working concentrations were: Yoda1 5, 10, 15 µM, as indicated; defactinib 2 µM, saracatinib 5 µM, and VEGF165 50 ng/mL. Concentrations were selected based on CCK-8 viability screening to avoid effects on proliferation/viability (see Additional file 1: Figure **S1**A–C). For VEGF165, the concentration was determined according to previous reports using the same dosage [10]. All compounds were freshly diluted into assay medium immediately before use. All control conditions included matched vehicle treatment (DMSO at the same final concentration as in Yoda1-treated conditions, ≤ 0.1%).

### Western blotting

Whole-cell lysates were prepared using RIPA buffer (Thermo Scientific). Protein concentration was determined using the BCA assay (Thermo Scientific). Equal amounts of protein were separated on 6–8% SDS-PAGE and transferred to PVDF membranes (Millipore, IPVH00010). Membranes were blocked in 0.3% BSA/TBS-T (30 min, RT), incubated with primary antibodies overnight at 4 °C, washed, and incubated with HRP-conjugated secondary antibodies for 1 h at room temperature (RT). Primary antibodies (typical dilutions): Src (CST #2108, 1:1000), p-Src Tyr416 (CST #2101, 1:1000), FAK (CST #3285, 1:1000), p-FAK Tyr397 (CST #3283, 1:1000), PYK2 (CST #3292, 1:1000), p-PYK2 Tyr402 (CST #3291, 1:1000), p-Occludin Tyr287 (Thermo #PA5-105344, 1:1000), p-Occludin Ser507 (Thermo #PA5-105057, 1:1000), p-Occludin Thr382 (Thermo #PA5-105056, 1:1000), Occludin (CST #91131, 1:1000), p-Paxillin Tyr118 (CST #69363, 1:1000), Paxillin (CST #12065, 1:1000), GAPDH (Santa Cruz sc-32233, 1:4000). Signals were developed using ECL reagents (Bio-Rad; Thermo Scientific high-sensitivity ECL) and detected with a LuminoGraph II system (ATTO, Tokyo, Japan). Band intensities were quantified using CS Analyzer 4 software (ATTO). When the same biological samples were analyzed across different gels, band intensities were first normalized to GAPDH within each gel to account for gel-to-gel variation. Phosphorylation levels were then expressed as phospho/total ratios using the corresponding total protein signals. Each data point represents an independent experiment. Saracatinib (5 µM) was added immediately before Yoda1 stimulation, whereas defactinib (2 µM) was added 12 h before Yoda1 stimulation.

### Real-time impedance assay (xCELLigence)

Impedance was measured using the xCELLigence Real-Time Cell Analysis System (ACEA Biosciences) with 16-well E-plates. The Cell Index (CI) was calculated as CI = (impedance at time point n - impedance in the absence of cells)/nominal impedance value [11]. Wells were gelatin-coated (≥ 45 min, 37 °C), equilibrated with 100 µL medium, and seeded at 20,000 cells/well. CI was acquired hourly until plateau. For barrier assays, cells were switched to DMEM/F-12 + 10% FBS without endothelial growth supplement. Yoda1 was used at 5, 10, or 15 µM in dose–response experiments. Yoda1 (15 µM) was added at time 0; saracatinib (5 µM) was added immediately before Yoda1; defactinib (2 µM) was added 12 h before Yoda1 under the same medium. After Yoda1 addition, impedance was sampled every 2 min. CI was normalized to the last pre-Yoda1 time point. Barrier disruption was quantified as the mean normalized CI during 10–20 min after Yoda1 addition (trough window). Within each experiment, multiple wells within the same E-plate were assigned to each treatment condition. Because each well contains a separate microelectrode, impedance was recorded independently for each well; however, wells within the same E-plate were considered technical replicates. The experiments were repeated in separate experimental runs on different occasions, and similar trends were observed.

### FITC–dextran permeability assay

HBEC-5i were seeded on ThinCert™ 24-well inserts (0.4 μm pore; Greiner #662640; growth area 0.33 cm²) with 200 µL apical / 600 µL basolateral volume and cultured to confluence (1–2 days). For dose–response experiments, HBEC-5i cells were treated with Yoda1 (5 or 15 µM) for 5 min. For inhibitor experiments, saracatinib (5 µM) was added immediately before stimulation with Yoda1 (15 µM). After stimulation, cells were washed with PBS, and FITC–dextran (4 kDa, Sigma #46944) at 1 mg/mL in DMEM/F-12 + 10% FBS was added apically (200 µL) for 20 min at 37 °C. Basolateral solutions were collected, transferred to black 96-well plates, and fluorescence was read (Ex 480 nm / Em 530 nm; PerkinElmer NIVO). Medium-only background was subtracted. Apparent permeability (P_app) was calculated as P_app = (dQ/dt) / (A × C₀), where C₀ is the initial apical concentration (µg/mL), A is the insert growth area (cm²), and dQ/dt is the average rate of FITC–dextran transport into the basolateral compartment from 0 to 20 min (µg/min), calculated as (Q₂₀ − Q₀)/20 min. The transported mass (Q) was determined using a standard curve (0–500 µg/mL) prepared in parallel.

### Immunocytochemistry

HBEC-5i (1.5 × 10⁴ cells/well) were plated on circular coverslips in 4-chamber slides pre-coated with gelatin (≥ 45 min, 37 °C). After reaching confluence, cells were stimulated with Yoda1 (15 µM, 20 min), then fixed with 4% paraformaldehyde (pH 7.4) for 10 min at 37 °C, washed (PBS ×3), permeabilized with 0.1% Triton X-100 (15 min, RT), and blocked in 2% BSA/PBS (60 min, RT). Primary staining was performed with ZO-1 (D6L1E) Rabbit Monoclonal Antibody (CST #13663, 1:200) overnight at 4 °C. After washing, cells were incubated with Alexa Fluor 488-conjugated goat anti-rabbit IgG (H + L), cross-adsorbed secondary antibody (Invitrogen #A-11008, 1:1000) for 1 h at room temperature. F-actin and nuclei were counterstained with rhodamine–phalloidin (Cytoskeleton PHDR1, 1:200) and DAPI (0.5–1 µg/mL), respectively. Images were acquired on a BZ-X800 microscope (Keyence) using identical exposure settings within an experiment. Immunocytochemical images of ZO-1 and F-actin were used for qualitative morphological assessment, and no quantitative image analysis was performed. Saracatinib (5 µM) was added immediately before Yoda1 stimulation, whereas defactinib (2 µM) was added 12 h before Yoda1 stimulation.

### Phosphoproteomic analysis

Phosphoproteomic analysis was performed as previously described [12]. HBEC-5i were treated with Yoda1 (15 µM, 1 min), with saracatinib (5 µM) added immediately before Yoda1 stimulation. Four biological replicates (*n* = 4) were prepared for each condition. After treatment, cells were washed with cold PBS, detached with Accutase, collected by centrifugation, and washed with cold PBS. The resulting cell pellet was resuspended in lysis buffer containing 50 mM NH₄HCO₃ and 8 M urea (100 µL per well), sonicated on ice, and centrifuged at 15,000 rpm for 10 min at 4 °C. The supernatant was collected, and the protein concentration was measured using the Bradford method.

100 µg of proteins were reduced with DTT (final concentration, 10 mM) at 37 °C for 60 min, followed by alkylation with iodoacetamide (final concentration, 25 mM) for 15 min at room temperature in the dark. The sample was then diluted ∼3-fold with 50 mM NH4HCO3 to reduce the urea concentration to below 2 M. Trypsin (Promega) was added at an enzyme-to-substrate ratio of 1:20 and digestion was performed at 37 °C overnight (∼16 h). After desalting using a C18 StageTip, the eluted peptides were lyophilized and stored at − 80 °C until use [12].

Phosphorylated peptide enrichment was carried out using a homemade TiO2-C8 tip column prepared by layering 3 mg of TiO2 beads (GL Sciences) on an Empore C8 disk filter (3 M) in a 200-µL pipette tip (D200, Gilson), according to the Titansphere Phos-TiO kit (GL Sciences) protocol. Phosphopeptides were eluted with an ammonia solution or pyrrolidine, acidified to pH < 3 with 0.1% TFA, and then further desalted using a C18 StageTip [13]. The eluted peptides were lyophilized and stored at − 80 °C until use.

The final peptide samples were dissolved in 0.1% formic acid/2% acetonitrile (ACN) and analyzed using an UltiMate 3000 ultrahigh-performance liquid chromatography (Thermo Fisher Scientific) system coupled to a Q Exactive HF mass spectrometer (Thermo Fisher Scientific) with slight modifications to a previously reported analysis [12]. The mobile phases were as follows: A, 0.1% formic acid/2% ACN; and B, 0.1% formic acid/95% ACN. A linear gradient from 2% to 33% B was applied for 150 min. Raw MS data files were processed using Progenesis QI for Proteomics software (version 4.2, Nonlinear Dynamics) for label-free quantitative analysis. Peptide-ion abundances were normalized using the default “Normalise to all proteins” procedure, which applies a run-specific global scaling factor calculated from peptide-ion abundance ratios relative to an automatically selected reference run. Protein identification was performed using the MASCOT search engine (version 3; Matrix Science, London, United Kingdom) against the human UniProtKB/Swiss-Prot (2024.06) database (http://www.uniprot.org/), and search parameters were in accordance with a previous study [13]. Peptides with a false discovery rate of < 1% and a Mascot ion score > 30 were retained for protein identification. Phosphosite localization was not independently assessed using a localization score or probability cutoff. Accordingly, all analyses were performed at the phosphopeptide-ion level rather than at the individual phosphosite level.

### Phosphoproteomic data analysis and functional enrichment

Normalized phosphopeptide-ion abundances were variance-stabilized in Progenesis QI using an inverse hyperbolic sine (arcsinh) transformation. CTRL versus Yoda1 and Yoda1 versus Yoda1 plus saracatinib were analyzed as separate two-group comparisons using one-way ANOVA under a between-subject experimental design. Normalized phosphopeptide-ion abundances and nominal ANOVA P values were exported from Progenesis QI for downstream analysis. Phosphopeptide-ion features quantified in at least 50% of samples were retained for downstream analysis. Per-feature z-scores were calculated for PCA and heatmap visualization. No missing-value imputation was performed. Log₂ fold changes were calculated from the mean normalized abundances of the two groups in each comparison.

Volcano plots (R v4.3.2, ggplot2) display log₂ fold change on the x-axis and − log₁₀(p) on the y-axis. Peptides observed only in Yoda1 (not CTRL) were assigned log₂FC = + Inf and clipped to + 10 for plotting. Differential phosphopeptides were screened using an exploratory threshold of an absolute log₂ fold change > 1 and a nominal ANOVA P value < 0.05. Multiple-testing correction was not applied at the phosphopeptide-selection stage. The resulting phosphoprotein lists were used for hypothesis-generating functional enrichment analyses. For GO, KEGG, and Reactome enrichment analyses, P values were adjusted using the Benjamini–Hochberg method, and an FDR < 0.05 was considered significant.

Genes were mapped via org.Hs.eg.db (SYMBOL→ENTREZ) and tested for GO Biological Process, Molecular Function, and Cellular Component enrichment using clusterProfiler::enrichGO. One representative phosphopeptide feature per gene (largest |log₂FC|, then lowest p if tied) was used. Enrichment p values were calculated by hypergeometric test and Benjamini–Hochberg adjusted; FDR < 0.05 was considered significant. KEGG and Reactome pathway enrichment were additionally assessed using enrichKEGG and enrichPathway, and visualized using the same parameters.

For network visualization, enriched terms (BP/CC/MF) were exported as a generic results table (GO ID, term name, p value, FDR, and associated genes) and loaded into Cytoscape (v3.10.3) with EnrichmentMap (v3.5.0). Networks were built using a p value cutoff of 0.05, FDR cutoff of 0.05, Combined coefficient similarity metric (Jaccard + Overlap), and edge cutoff 0.25. The Top-50 terms by FDR were visualized.

### Animals and surgery

Male C57BL/6J mice (10 weeks; 24–27 g; Japan SLC) were anesthetized with inhaled isoflurane (induction 3–4% and maintenance 2–3% in oxygen via a nose cone), delivered continuously throughout the non-recovery surgical procedure, and maintained at approximately 37 °C on a heating pad maintained at a constant temperature. Bilateral common carotid artery occlusion (BCCAO) was induced via midline neck incision, isolation of both CCAs, and ligation with 7 − 0 silk for 10 min, followed by release of the ligature to allow reperfusion. GsMTx4 (1 mg/kg, i.p.) was administered 1.5 h before occlusion. Five minutes after reperfusion, FITC–dextran 4 kDa (80 mg/kg; 2 mg in 100 µL sterile PBS) was injected into the subclavian vein (30G needle). Ten minutes later, mice were perfused transcardially with heparinized saline (150 IU/mL) for 3 min; brains were harvested, embedded in OCT, snap-frozen in 2-methylbutane (Tokyo Chemical Industry M0167) cooled to − 30 to − 50 °C, and cryosectioned at 10 μm. Pre-specified exclusion criteria included major bleeding, or failed venous cannulation. A total of 12 mice were included in the FITC–dextran permeability experiment (*n* = 4 per group), and no animals met the exclusion criteria or were excluded from the final analysis. Adequacy of anesthesia was ensured by observing the absence of spontaneous movement and stable respiratory patterns throughout the procedure. Because this was a non-recovery procedure performed entirely under continuous isoflurane anesthesia and immediately followed by transcardial perfusion, no additional analgesic was administered. Euthanasia at the end of the experiments was achieved by transcardial perfusion with heparinized saline under deep isoflurane anesthesia (4–5%), followed by bilateral thoracotomy and confirmation of cardiac arrest. For animals that met the pre-specified exclusion criteria, euthanasia was performed by carbon dioxide (CO₂) inhalation in a dedicated chamber, administered at a displacement rate of 20–30% of chamber volume per minute until respiratory arrest was observed; death was confirmed by the absence of spontaneous respiration and detectable heartbeat. Only male mice were used in this study to minimize potential variability related to sex hormones and to align with the majority of prior ischemia–reperfusion studies; sex-specific effects were not assessed. Mice were allocated to the experimental groups before surgery without a formal randomization procedure. All procedures were approved by the institutional committee (F-A-24-059).

### Cerebral blood flow imaging

After scalp removal and skull exposure, cortical blood flow was recorded using an OMEGAZONE OZ-2mini laser tissue blood-flow imager (Muromachi Kikai). Symmetric circular ROIs (2 mm diameter) were placed over parietal cortex at 2 mm lateral and 3 mm caudal to bregma. Cerebral blood flow (CBF) was recorded at baseline before occlusion (Pre), immediately after BCCAO, and immediately after the ligatures were released following 10 min of occlusion to initiate reperfusion (Post). At each time point, 10 consecutive frames were acquired and averaged. CBF was normalized to the Pre value within each animal.

### Vascular permeability quantification

To minimize intravascular background, mice were transcardially perfused with heparinized saline before tissue collection. A single coronal section at approximately bregma − 2.7 mm (interaural + 1.0 mm) was imaged under identical exposure and gain settings using a BZ-X800 fluorescence microscope (Keyence). In each hemisphere, one cortical region at the same anatomical level was analyzed. Fluorescence intensity of 4 kDa FITC–dextran was quantified as mean fluorescence intensity per unit area using the built-in analysis software (BZ-X Analyzer, Keyence). Identical measurement parameters were applied to all images. Analysis was performed with pre-specified parameters and by an investigator blinded to group allocation.

### Statistics

Data are presented as mean ± SD. Statistical analysis was performed in GraphPad Prism 10.6.1 (GraphPad Software Inc., San Diego, CA, USA). Unless otherwise stated, n represents the number of independent biological experiments or individual animals. After confirming normality, two-tailed unpaired Student’s t-tests were used for two-group comparisons. Pearson’s correlation was used to assess associations between continuous variables. Comparisons among three or more groups were performed using one-way ANOVA followed by Tukey’s multiple comparisons test. For dose-response experiments involving comparisons with a single control group, one-way ANOVA followed by Dunnett’s multiple comparisons test was used.

For impedance-based barrier assays, barrier disruption was quantified as the mean normalized CI during 10–20 min after Yoda1 addition. For each independent xCELLigence experimental run, technical-replicate wells within each condition were averaged to obtain one value per condition. Comparisons between CTRL and Yoda1 (15 µM) and between Yoda1 and Yoda1 plus saracatinib across independent experimental runs were performed using paired two-tailed Student’s t-tests.

For CBF, a two-way repeated-measures ANOVA (treatment × time) with Geisser–Greenhouse correction was applied; Sidak’s multiple comparisons test was used for between-group comparisons at each time point, and Dunnett’s test for within-group comparisons versus baseline (Pre). Significance was defined as *p* < 0.05. All p values and n are reported in the figure legends.

### Use of AI-assisted language editing

During the preparation of this manuscript, the authors used ChatGPT (OpenAI) to assist with English language editing and proofreading. The authors reviewed and edited the output as needed and take full responsibility for the content of the manuscript.

## Results

To investigate the effects of Piezo1 activation on brain endothelial signaling, we performed a phosphoproteomic analysis using the Piezo1 agonist Yoda1 [13]. HBEC-5i were stimulated with Yoda1 for 1 min (Yoda1 group) or with matched vehicle (CTRL group), and their phosphoproteomic profiles were compared.

**Fig. 1:**
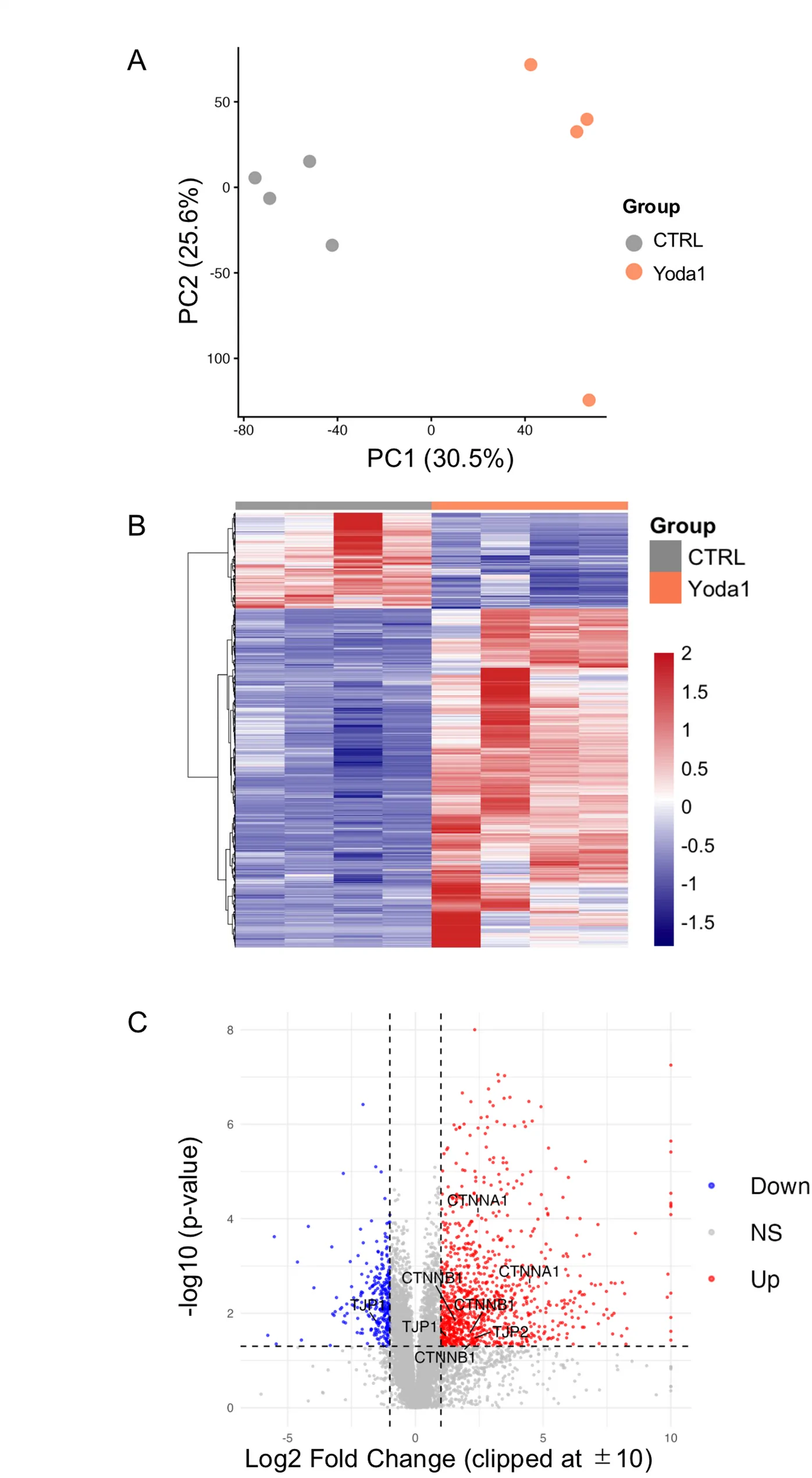
Piezo1 activation broadly alters the phosphoproteomic profile of brain endothelial cells. **(A)** Principal component analysis (PCA) of phosphoproteomic profiles in human brain microvascular endothelial cells (HBEC-5i) treated with the Piezo1 agonist Yoda1 (15 µM, 1 min) or vehicle (CTRL). Four independent biological replicates were analyzed per condition (*n* = 4). **(B)** Heatmap of phosphopeptide-ion features meeting the exploratory criteria showing an overall increase in phosphorylation after Yoda1 stimulation. **(C)** Volcano plot of differential phosphorylation. Red and blue dots indicate phosphopeptide-ion features meeting the exploratory criteria for increased or decreased abundance after Yoda1 treatment, respectively. Increased phosphorylation was observed in junction-related proteins, including CTNNA1, CTNNB1, TJP1, and TJP2

Principal component analysis (PCA) of all detected phosphopeptides revealed a clear separation between the CTRL and Yoda1 groups (Fig. 1A), indicating that Yoda1 stimulation induced extensive phosphorylation changes across the phosphoproteome. A heatmap of phosphopeptide-ion features meeting the exploratory criteria showed coordinated differences in phosphorylation patterns between CTRL and Yoda1-treated cells (Fig. 1B). Overall phosphorylation levels tended to increase in the Yoda1 group, suggesting a global shift toward enhanced phosphorylation activity. Consistent results were obtained in the volcano plot analysis (Fig. 1C), which showed more phosphopeptide-ion features meeting the exploratory criteria for increased than decreased abundance after Yoda1 treatment. These findings suggest an overall shift toward increased phosphorylation-associated signals following Piezo1 activation. Notably, several phosphorylated proteins were associated with cell–cell junctions, including those involved in adherens and tight junction complexes.

**Fig. 2:**
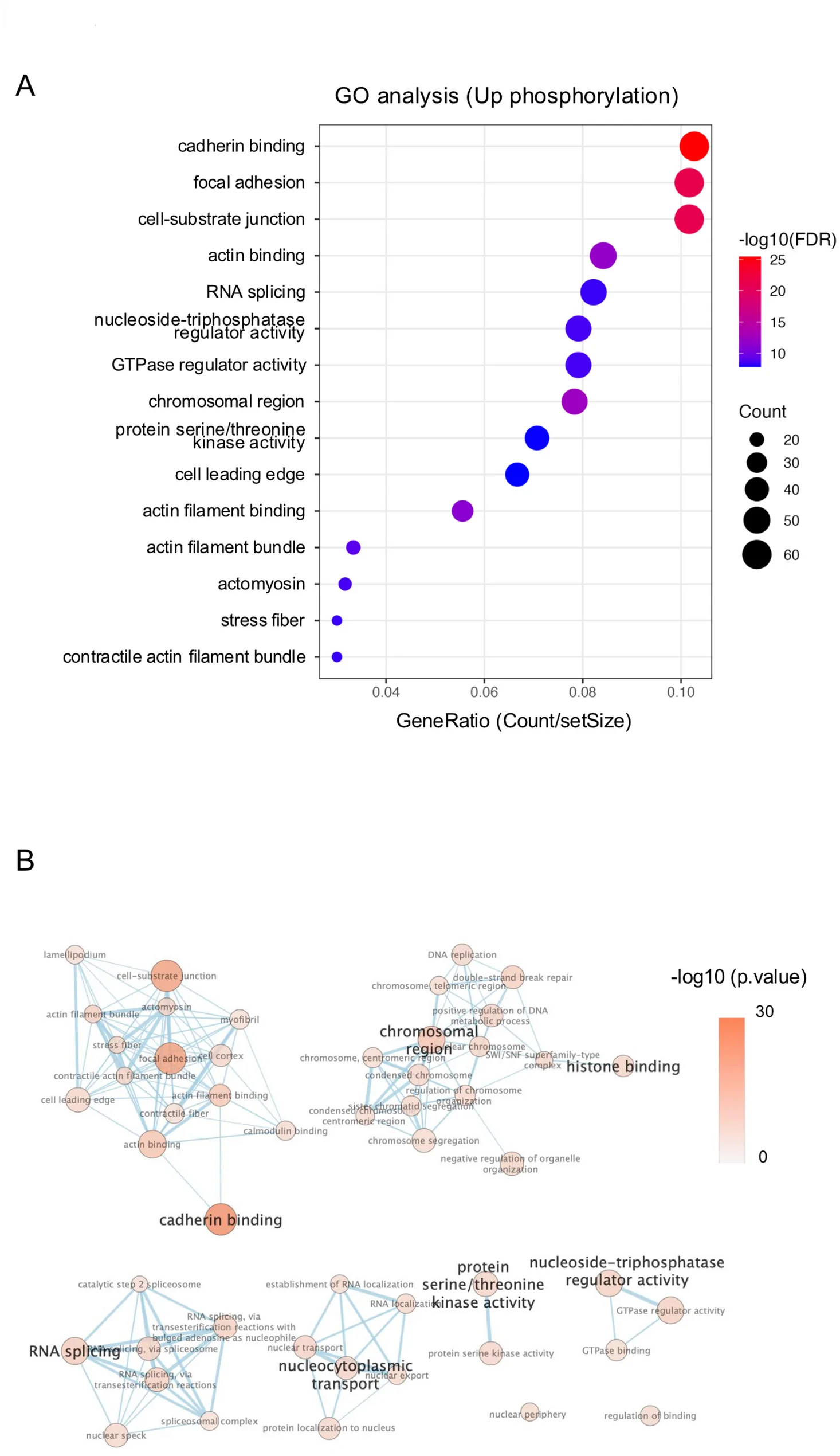
Piezo1 activation induces phosphorylation changes in junctional and cytoskeletal pathways. **(A)** Gene Ontology (GO) enrichment analysis of proteins with increased phosphorylation (top 15 terms ranked by FDR). Enriched terms included cadherin binding, focal adhesion, cell-substrate junction, and actin binding, indicating junctional and cytoskeletal remodeling. The x-axis shows GeneRatio (Count/setSize); dot size indicates protein count; dot color represents –log10(FDR). **(B)** Network visualization of enriched GO terms. In addition to junction- and cytoskeleton-related categories, nuclear processes such as RNA splicing, chromosomal region, and nucleocytoplasmic transport were also enriched. Node size indicates the number of genes per set; node color represents enrichment score (–log10 *P* value); edge width indicates gene overlap

Gene Ontology (GO) enrichment analysis revealed that proteins exhibiting increased phosphorylation upon Yoda1 stimulation were significantly enriched in terms related to cell–cell junctions and cell adhesion, such as cadherin binding, focal adhesion, and cell–substrate junction (Fig. 2A; see Additional file 3: Table **S1**). Moreover, GO categories associated with the actin cytoskeleton—including actin binding, actin filament bundle, and stress fiber—were among the top enriched terms, suggesting that Piezo1 activation contributes to actin cytoskeletal remodeling and junctional reorganization.

Enrichment in nucleoside-triphosphatase regulator activity and GTPase regulator activity further implied potential involvement of Rho family GTPases in these phosphorylation-dependent processes. Furthermore, Kyoto Encyclopedia of Genes and Genomes (KEGG) and Reactome pathway analyses identified focal adhesion, adherens junction, and regulation of actin cytoskeleton among the top enriched pathways, along with the Rho GTPase cycle (Rac1/RhoA/CDC42) (see Additional file 1: Figures **S2**A–B**; **Additional file 4: Table **S2****; **Additional file 5: Table **S3**), clearly indicating alterations in phosphorylation events associated with Rho GTPase signaling. All of these pathways are deeply involved in the maintenance of endothelial barrier integrity [14].

Conversely, proteins with decreased phosphorylation following Yoda1 stimulation were significantly enriched in terms associated with microtubule/tubulin binding, GTPase regulator/activator activity, and chromosomal or centromeric regions (see Additional file 1: Figures **S3**A–B; Additional file 6: Table **S4**). These results suggest that Piezo1 activation may suppress microtubule-related phosphorylation events while enhancing actin–adhesion signaling. Collectively, these enrichment results suggest that Yoda1-responsive phosphorylation changes are associated with actin- and junction-related processes, together with decreased phosphorylation of microtubule-associated proteins. These patterns may be relevant to the observed endothelial barrier impairment. Finally, network visualization identified functionally related GO terms spanning junctional/cytoskeletal and nuclear processes (Fig. 2B). These enrichment patterns are hypothesis-generating and indicate biological processes associated with Yoda1-responsive phosphorylation changes rather than direct pathway activation.

Phosphoproteomic analysis revealed that Piezo1 activation markedly altered phosphorylation events associated with cell adhesion, cytoskeletal remodeling, and Rho GTPase signaling. These pathways—focal adhesion, adherens junction, Rho GTPase cycle, and actin cytoskeleton regulation- are closely linked to the maintenance of endothelial barrier integrity [14], and Src family kinases (SFKs) are known to play pivotal upstream regulatory roles [15, 16]. SFKs phosphorylate FAK and its homologue PYK2, thereby promoting focal-adhesion assembly and activation of Rho GTPases, which together drive actin stress-fiber formation and junctional remodeling [17]. Previous studies have shown that SFK–FAK/PYK2 signaling regulates focal-adhesion components such as paxillin and contributes to the phosphorylation-dependent modulation of endothelial barrier integrity [7, 18]. These findings suggested that Piezo1 activation may modulate endothelial barrier function through SFK–FAK/PYK2–Rho GTPase signaling. To examine this hypothesis, HBEC-5i cells were stimulated with the Piezo1 agonist Yoda1, and Src, FAK/PYK2 and paxillin activation as well as changes in tight junction structure were evaluated.

**Fig. 3:**
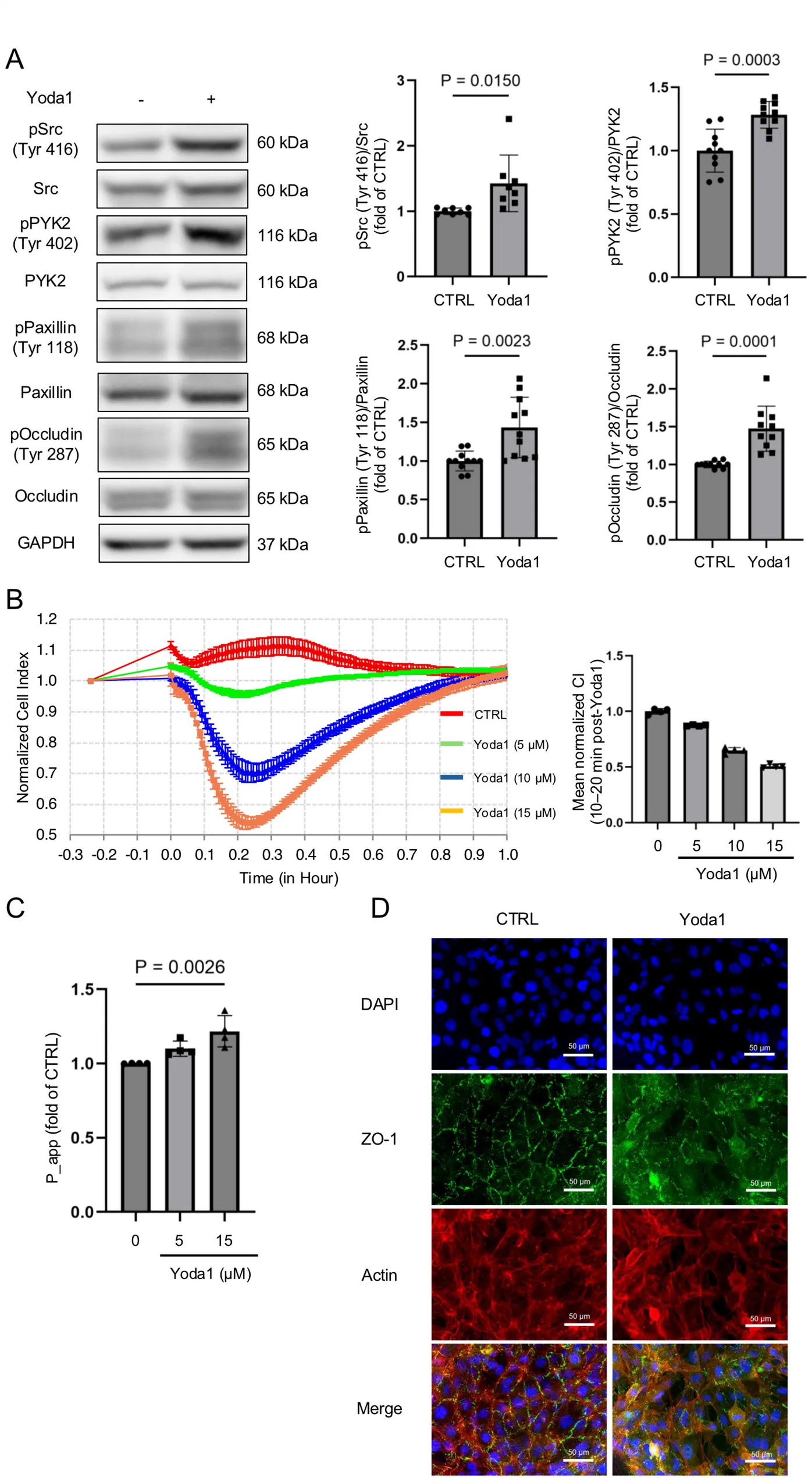
Piezo1 activation induces occludin phosphorylation and rapidly disrupts tight junction integrity. **(A)** HBEC-5i cells were stimulated with the Piezo1 agonist Yoda1 (15 µM, 1 min), and phosphorylation of Src-related and tight junction-associated proteins was assessed. Data are mean ± SD; *n* indicates independent experiments. Two-tailed unpaired Student’s *t*-test: pSrc/Src (*n* = 8, *P* = 0.015), pPYK2/PYK2 (*n* = 10, *P* = 0.0003), pPaxillin/Paxillin (*n* = 11, *P* = 0.0023), and pOccludin/Occludin (*n* = 10, *P* = 0.0001). **(B)** Real-time impedance analysis (xCELLigence), as assessed by the Cell Index, showed a concentration-dependent decrease in cell impedance after Yoda1 stimulation (5, 10, or 15 µM), indicating impaired barrier integrity. Right, mean normalized Cell Index during 10–20 min after Yoda1 addition (trough window), normalized to the last pre-Yoda1 time point. Data are presented as mean ± SD of four technical-replicate wells per condition within a single E-plate from one representative experiment. Lower values indicate greater barrier disruption. The CTRL versus Yoda1 15 µM comparison was reproduced in seven independent experimental runs, and the full dose-response experiment including 5, 10, and 15 µM Yoda1 was independently performed twice, with similar results. Statistical analyses across independent experimental runs are shown in Additional file 1: Figure S5A. **(C)** A 4 kDa FITC-dextran permeability assay showed increased paracellular permeability after Yoda1 treatment (5 or 15 µM). Data are mean ± SD from four independent biological experiments (*n* = 4). Higher values indicate greater permeability. One-way ANOVA with Dunnett’s test versus CTRL: 5 µM, *P* = 0.12; 15 µM, *P* = 0.0026. **(D)** Representative immunofluorescence images of ZO-1 (green) and F-actin (red) in control and Yoda1-treated cells. Scale bars, 50 μm

Yoda1 stimulation for 1 min induced phosphorylation of occludin at Tyr287, a key tight junction component (Fig. 3A) [19]. Increased phosphorylation was also observed for Src and adhesion-related proteins PYK2 and paxillin, whereas phosphorylation of occludin at Thr382 or Ser507 remained unchanged (see Additional file 1: Figure **S4**A). FAK phosphorylation was also unchanged (see Additional file 1: Figure **S4**B). Because PYK2 and paxillin function downstream of Src within focal adhesion and junctional signaling pathways, their phosphorylation suggests activation of Src-mediated adhesion-associated kinase cascades in response to Piezo1 stimulation. Functional assays demonstrated that Yoda1 caused a dose-dependent disruption of tight junction integrity as measured by real-time impedance monitoring using the xCELLigence system (Fig. 3B). Statistical analysis of the seven independent experimental runs is shown in Additional file 1: Figure S5A. Similarly, FITC–dextran permeability assays showed a significant increase in paracellular permeability (Fig. 3C). Fluorescent staining of the tight-junction protein ZO-1 and filamentous actin (F-actin) revealed continuous junctional localization in control cells, whereas Yoda1-treated cells exhibited cytoskeletal elongation, intercellular gap formation, and junctional discontinuity (Fig. 3D). Live-cell imaging further visualized the progressive disruption of intercellular junctions, cell contraction, and cytoskeletal reorganization following Yoda1 stimulation (see Additional file 9: Supplementary Movie 1; Additional file 10: Supplementary Movie 2).

These findings are consistent with the GO enrichment results highlighting cadherin binding, focal adhesion, and actin binding categories, supporting the notion that Piezo1 activation induces structural and functional remodeling of the cytoskeleton that compromises cell adhesion and intercellular junctions. Together, these findings indicate that Piezo1 activation promotes occludin Tyr287 phosphorylation and rapid disruption of tight junction integrity in brain microvascular endothelial cells.

**Fig. 4:**
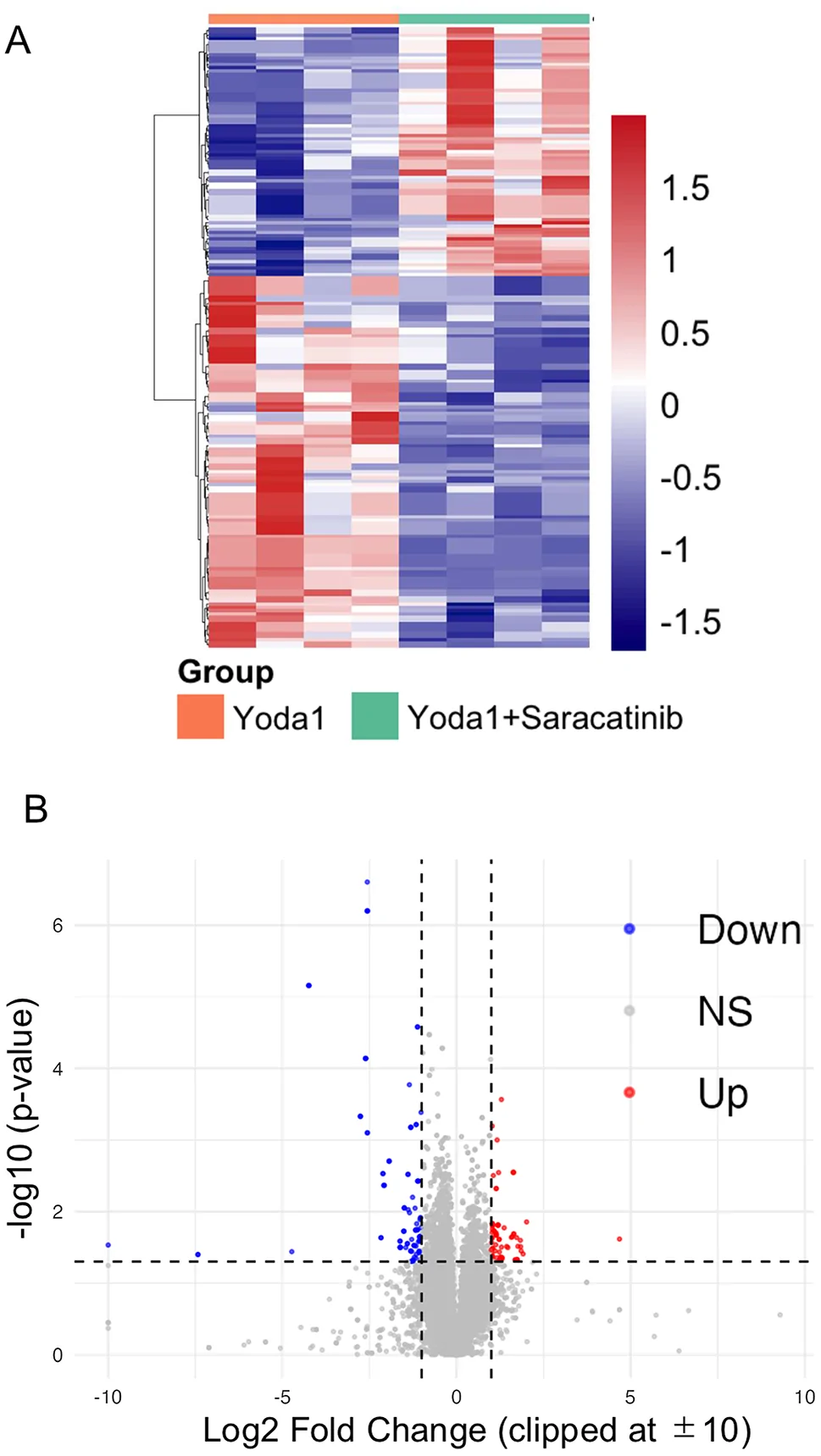
Saracatinib-sensitive phosphorylation changes following pharmacological Piezo1 activation. **(A)** Heatmap showing hierarchical clustering of phosphopeptide-ion features meeting the exploratory criteria in the comparison between Yoda1-stimulated cells (15 µM, 1 min) and cells co-treated with Yoda1 and saracatinib (5 µM). Four independent biological replicates were analyzed per condition (*n* = 4). **(B)** Volcano plot comparing phosphopeptide-ion features meeting the exploratory criteria for increased or decreased abundance in the Yoda1 plus saracatinib group compared with the Yoda1 group, respectively

The above findings indicated that Piezo1 activation disrupts endothelial barrier integrity in brain microvascular endothelial cells and implicated Src signaling in this process. To further examine this mechanism, phosphoproteomic analysis was performed in HBEC-5i cells stimulated with the Piezo1 agonist Yoda1 (Yoda1 group) and in those co-treated with the Src inhibitor saracatinib (Yoda1 + saracatinib group). PCA of all detected phosphopeptides showed no clear separation between the Yoda1 and Yoda1 + saracatinib groups (see Additional file 1: Figure **S6**). However, heatmap visualization of phosphopeptide-ion features meeting the exploratory criteria suggested attenuation of phosphorylation changes in the presence of saracatinib (Fig. 4A). Consistently, volcano plot analysis showed relative decreases in phosphopeptide-ion abundance in the Yoda1 plus saracatinib group compared with the Yoda1 group (Fig. 4B).

**Fig. 5:**
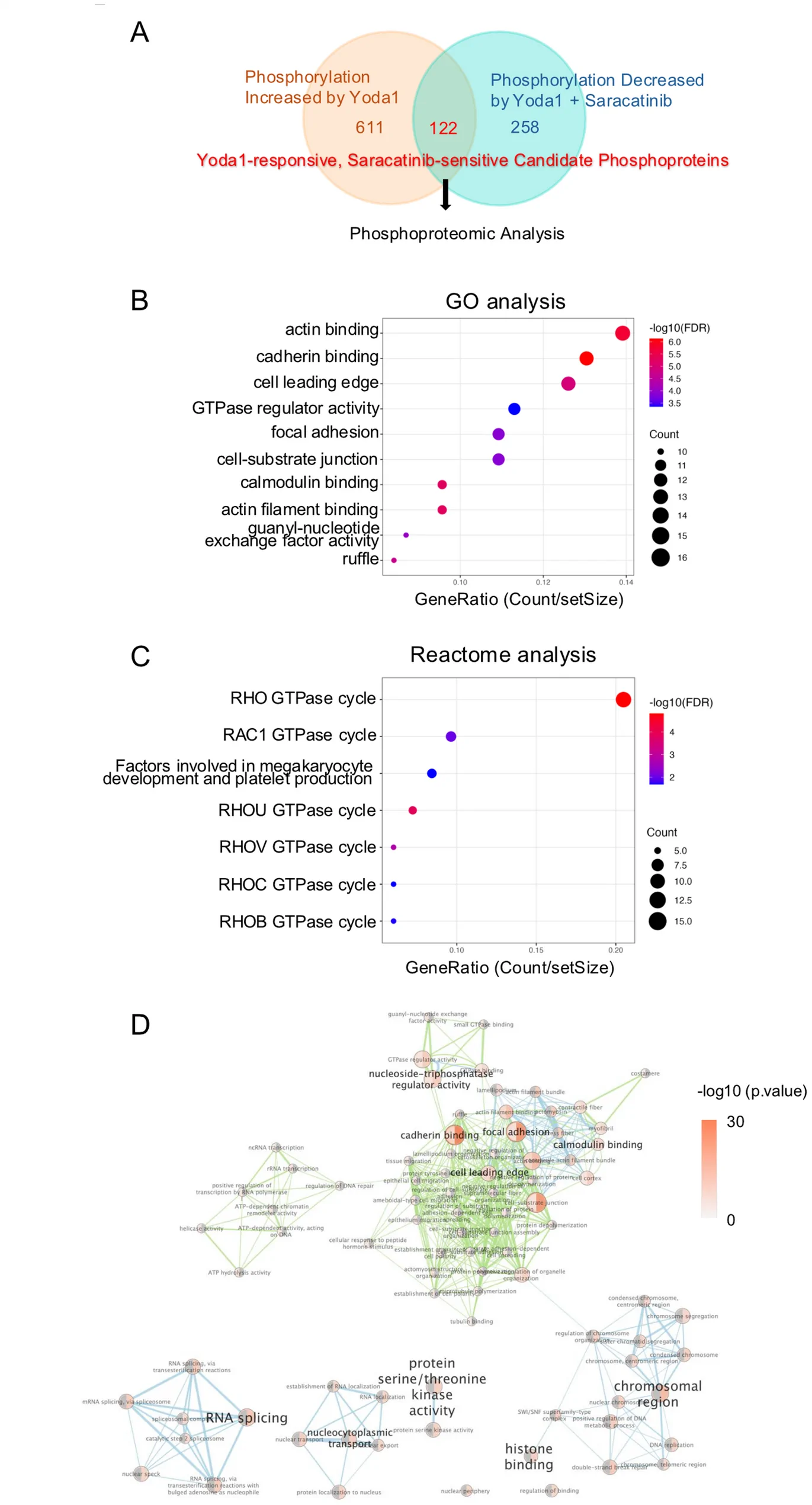
Saracatinib-sensitive phosphorylation following Piezo1 activation is enriched in junctional and cytoskeletal pathways. **(A)** Venn diagram showing the overlap between phosphopeptide-ion features increased after Yoda1 treatment and decreased after Yoda1 plus saracatinib treatment according to the exploratory criteria. A total of 122 phosphoproteins were identified as Yoda1-responsive, saracatinib-sensitive phosphoproteins and subjected to enrichment analysis. **(B)** Gene Ontology (GO) enrichment analysis of these 122 saracatinib-sensitive phosphoproteins (top 10 terms ranked by FDR) showed enrichment for actin binding, cadherin binding, cell leading edge, and focal adhesion, indicating cytoskeletal and junctional regulation. **(C)** Reactome pathway enrichment analysis (top 10 terms ranked by FDR) identified the Rho GTPase cycle as the top enriched pathway. **(D)** Functional network visualization based on GO enrichment terms. Saracatinib-sensitive phosphoproteins were enriched in terms related to cadherin binding, focal adhesion, and the cell leading edge. Green edges indicate saracatinib-sensitive networks, and blue edges indicate the broader Yoda1-responsive network

To identify saracatinib-sensitive phosphorylation events during Piezo1 activation, we selected 122 phosphoproteins represented by phosphopeptide-ion features that met the exploratory criteria for increased phosphorylation after Yoda1 treatment and decreased phosphorylation after Yoda1 plus saracatinib treatment (Fig. 5A), and subjected them to GO and Reactome enrichment analysis (see Additional file **7**: Table **S5**; Additional file **8**: Table **S6**). Enriched GO terms included actin binding, cadherin binding, cell leading edge, and focal adhesion, indicating that many of the phosphorylation events related to cell adhesion and cytoskeletal organization induced by Piezo1 are sensitive to saracatinib treatment (Fig. 5B). Reactome analysis revealed that the Rho GTPase cycle was the top enriched pathway (Fig. 5C). Collectively, these results suggest that, in brain microvascular endothelial cells, Piezo1-induced, saracatinib-sensitive phosphorylation changes were enriched in pathways related to intercellular junctions, cell adhesion, cytoskeletal remodeling, and Rho GTPase signaling.

Network analysis further demonstrated that, while Yoda1 stimulation broadly influenced multiple cellular processes-including nuclear functions (chromosomal regulation, RNA processing, and nucleocytoplasmic transport) and cytoskeletal pathways (cadherin binding and focal adhesion), as shown in Fig. 2B-the saracatinib-sensitive Piezo1 signaling primarily converged on cell–cell junctions, cell adhesion, and cytoskeletal remodeling (Fig. 5D). These results suggest that Src-family kinase activity may contribute to the phosphorylation changes associated with Yoda1 stimulation that regulate endothelial adhesive structures and barrier integrity.

Phosphoproteomic comparison between Yoda1-stimulated cells (Yoda1 group) and those co-treated with the Src inhibitor saracatinib (Yoda1 + saracatinib group) suggested that the disruption of endothelial barrier integrity induced by Piezo1 activation is sensitive to Src-family kinase inhibition. To further examine this association, we examined whether saracatinib treatment could prevent Yoda1-responsive tight junction dysfunction.

**Fig. 6:**
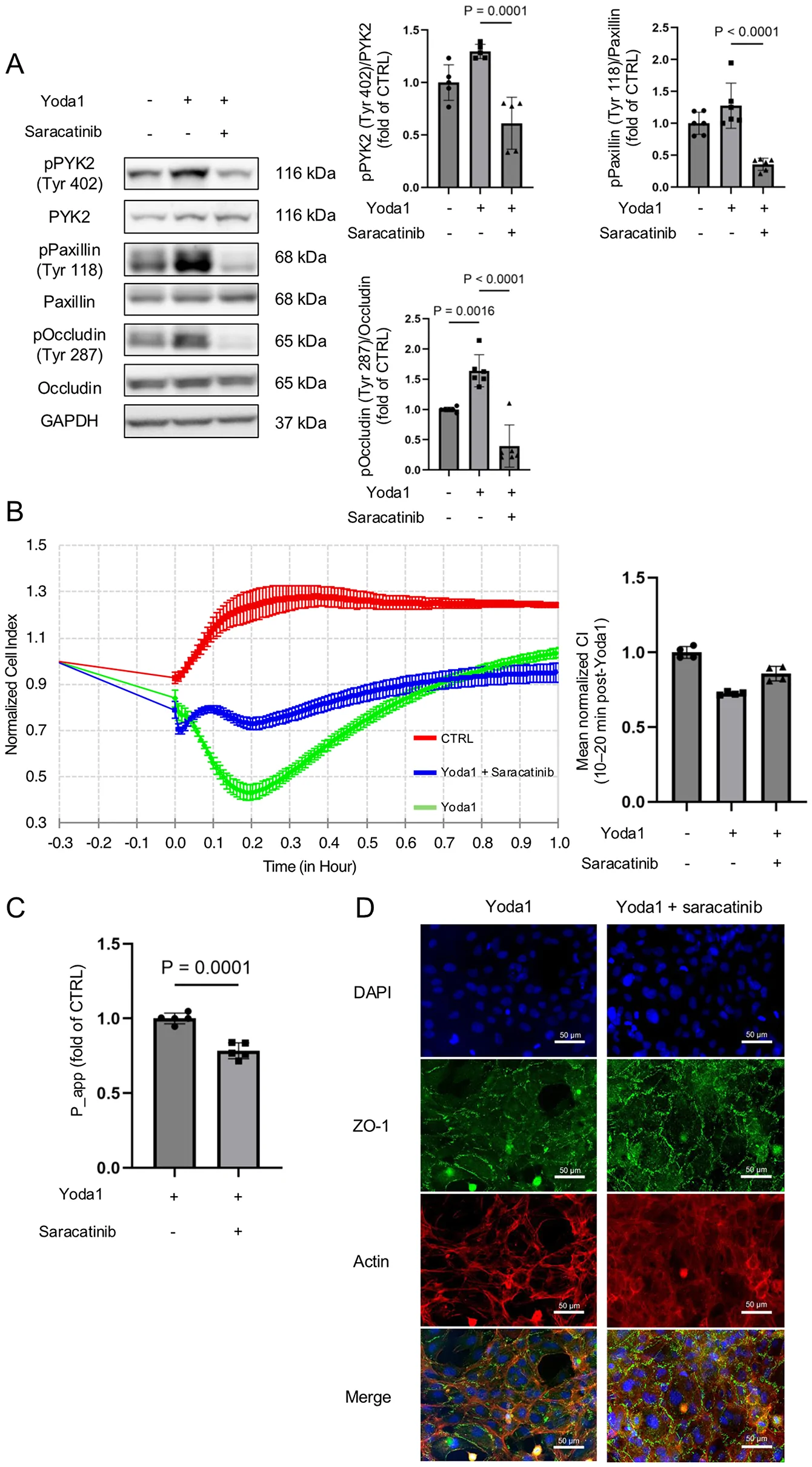
Saracatinib attenuates Piezo1-responsive phosphorylation and endothelial barrier disruption. **(A)** HBEC-5i cells were stimulated with Yoda1 (15 µM, 1 min) with or without saracatinib (5 µM). Phosphorylation of PYK2 (Tyr402), paxillin (Tyr118), and occludin (Tyr287) was analyzed by Western blot. Data are mean ± SD; *n* indicates independent experiments. One-way ANOVA with Tukey’s test: pPYK2/PYK2 (*n* = 5), CTRL vs. Yoda1, *P* = 0.0527; Yoda1 vs. Yoda1 + saracatinib, *P* = 0.0001; CTRL vs. Yoda1 + saracatinib, *P* = 0.0123. pPaxillin/Paxillin (*n* = 5), CTRL vs. Yoda1, *P* = 0.1366; Yoda1 vs. Yoda1 + saracatinib, *P* < 0.0001; CTRL vs. Yoda1 + saracatinib, *P* = 0.0007. pOccludin/Occludin (*n* = 6), CTRL vs. Yoda1, *P* = 0.0016; Yoda1 vs. Yoda1 + saracatinib, *P* < 0.0001; CTRL vs. Yoda1 + saracatinib, *P* = 0.0025. **(B)** Real-time impedance monitoring (xCELLigence) showed that saracatinib attenuated Yoda1-responsive barrier disruption. Right, mean normalized Cell Index during 10–20 min after Yoda1 addition (trough window), normalized to the last pre-Yoda1 time point. Data are mean ± SD of four technical-replicate wells per condition from one representative E-plate. The Yoda1 versus Yoda1 plus saracatinib comparison was reproduced in three independent experimental runs, and the statistical analysis across these runs is shown in Additional file 1: Figure S5B. Lower values indicate greater barrier disruption. **(C)** FITC-dextran (4 kDa) permeability assay showing that saracatinib attenuated Yoda1-responsive paracellular permeability. Data are mean ± SD (*n* = 5). Higher values indicate greater permeability. Two-tailed unpaired Student’s *t*-test: Yoda1 vs. Yoda1 + saracatinib, *P* = 0.0001. **(D)** Representative immunofluorescence images of ZO-1 (green) and F-actin (red) in Yoda1-treated cells and Yoda1 + saracatinib-treated cells. Scale bars, 50 μm

Western blot analysis revealed that Yoda1-responsive phosphorylation of PYK2, paxillin, and occludin was significantly suppressed by saracatinib treatment (Fig. 6A). Functional assessment using the xCELLigence real-time impedance system showed that Yoda1-responsive impairment of tight junction integrity was markedly attenuated in the presence of saracatinib (Fig. 6B). Statistical analysis of the three independent experimental runs is shown in Additional file 1: Figure S5B. Similarly, FITC–dextran permeability assays showed that saracatinib significantly reduced paracellular permeability under Yoda1 stimulation (Fig. 6C). Immunofluorescence staining of ZO-1 and F-actin revealed that cytoskeletal deformation, intercellular gap formation, and junctional disruption observed in Yoda1-treated cells were substantially prevented by saracatinib (Fig. 6D). Consistent with these findings, live-cell imaging demonstrated that saracatinib inhibited Yoda1-responsive cell contraction and junctional disassembly (see Additional file 11: Supplementary Movie 3).

Collectively, these results indicate that Piezo1 activation impairs tight junction function and endothelial barrier integrity in association with Src-family kinase-sensitive signaling and PYK2 phosphorylation, supporting the involvement of Yoda1-responsive, saracatinib-sensitive signaling in the regulation of tight junction dynamics in brain microvascular endothelial cells.

**Fig. 7:**
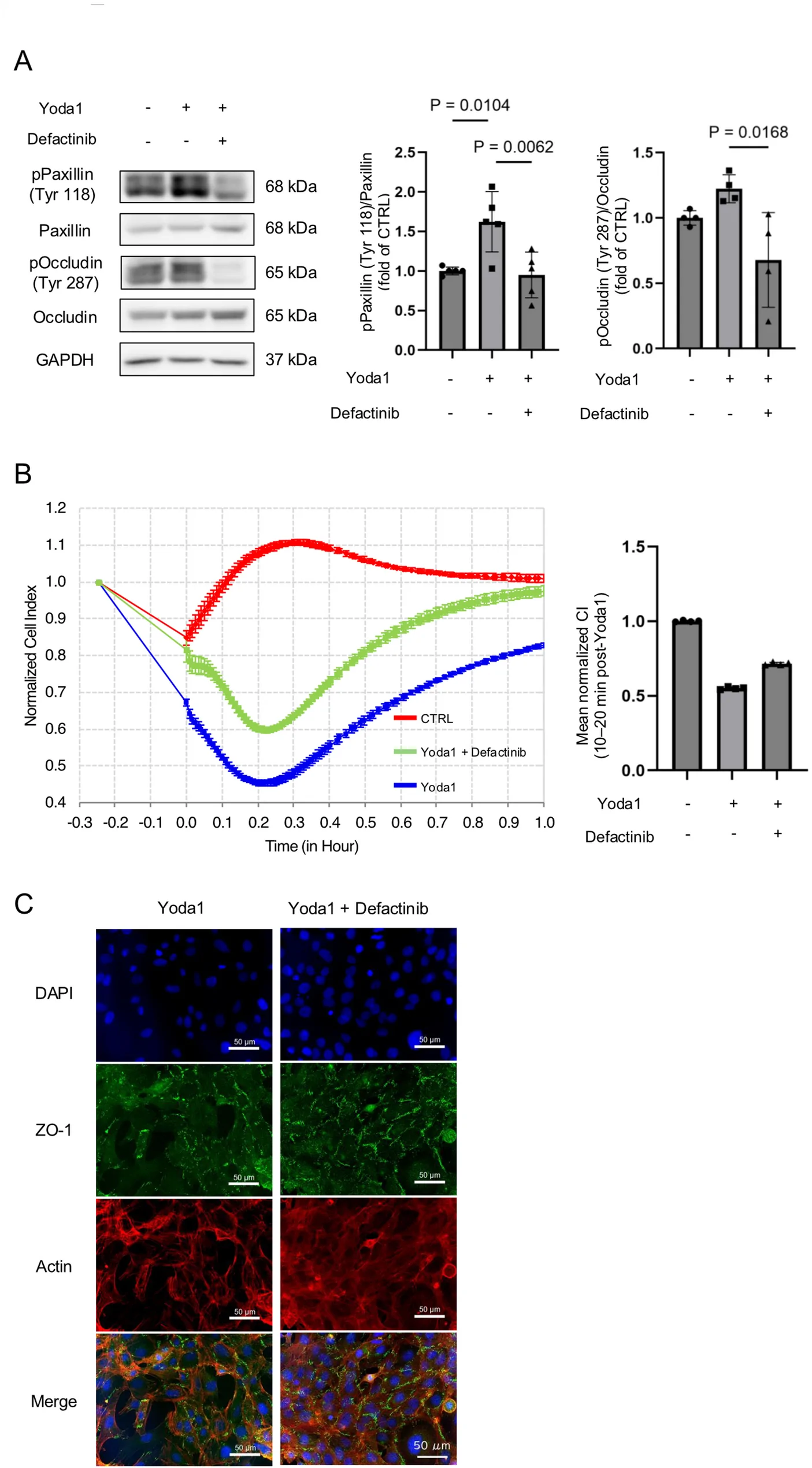
Defactinib attenuates Piezo1-responsive phosphorylation and endothelial barrier disruption. **(A)** HBEC-5i cells were stimulated with Yoda1 (15 µM, 1 min) with or without the PYK2/FAK inhibitor defactinib (2 µM), and phosphorylation of paxillin and occludin was assessed by Western blot. Data are mean ± SD; *n* indicates independent experiments. One-way ANOVA with Tukey’s test: pPaxillin/Paxillin (*n* = 5), CTRL vs. Yoda1, *P* = 0.0104; Yoda1 vs. Yoda1 + defactinib, *P* = 0.0062; CTRL vs. Yoda1 + defactinib, *P* = 0.9549. pOccludin/Occludin (*n* = 4), CTRL vs. Yoda1, *P* = 0.3658; Yoda1 vs. Yoda1 + defactinib, *P* = 0.0168; CTRL vs. Yoda1 + defactinib, *P* = 0.1527. **(B)** Real-time impedance monitoring (xCELLigence) showed that defactinib attenuated Yoda1-responsive barrier disruption. Right, mean normalized Cell Index during 10–20 min after Yoda1 addition (trough window), normalized to the last pre-Yoda1 time point. Data are mean ± SD of four wells per condition within a single E-plate (technical replicates) (*n* = 4 wells per condition within one E-plate). No inferential statistical analysis across independent experimental runs was performed. Lower values indicate greater barrier disruption. **(C)** Representative immunofluorescence images of ZO-1 (green) and F-actin (red) in Yoda1-treated cells and Yoda1 + defactinib-treated cells. Scale bars, 50 μm

To further examine defactinib-sensitive signaling, we examined the relationship among PYK2, paxillin, and occludin phosphorylation using the PYK2/FAK inhibitor defactinib. Western blot analysis revealed that Yoda1-responsive phosphorylation of paxillin and occludin was significantly suppressed by defactinib, indicating that defactinib attenuates the Piezo1-induced phosphorylation cascade (Fig. 7A).　Functional assessment of tight junction integrity using the xCELLigence real-time impedance system demonstrated that the Yoda1-responsive decrease in barrier resistance was attenuated by defactinib (Fig. 7B). Consistent results were obtained by immunofluorescence staining of ZO-1 and F-actin, which showed that cytoskeletal deformation, intercellular gap formation, and junctional disruption observed after Yoda1 stimulation were attenuated by defactinib treatment (Fig. 7C).

Collectively, these findings suggest that defactinib-sensitive signaling contributes to Piezo1-responsive phosphorylation changes and endothelial barrier disruption.

In vitro experiments suggested that Piezo1 activation contributes to the disruption of endothelial barrier integrity in brain microvascular endothelial cells. However, it remains unclear whether pharmacological activation by Yoda1 adequately reflects the abrupt hemodynamic changes that occur during reperfusion in vivo. Therefore, we hypothesized that abrupt hemodynamic changes at reperfusion engage Piezo1-associated signaling in vivo and examined whether BBB permeability increases during the ultra-acute phase after flow restoration. Furthermore, we tested whether GsMTx4 could attenuate this early increase in vascular permeability.

In typical murine and rat cerebral ischemia-reperfusion models, middle cerebral artery occlusion is performed for 30–90 min followed by several hours to days of reperfusion, whereas BCCAO is used for transient global ischemia followed by reperfusion over similar delayed time courses [20, 21]. These protocols are based on the established concept that inflammatory and protease-dependent structural disruption of endothelial junctions becomes apparent in the delayed phase after reperfusion [2, 22]. Consequently, permeability assessments are usually conducted several hours to one day after reperfusion. Although several studies have reported BBB hyperpermeability within tens of minutes after reperfusion [1, 23, 24], the upstream molecular mechanisms of this ultra-acute phase remain poorly defined.

**Fig. 8:**
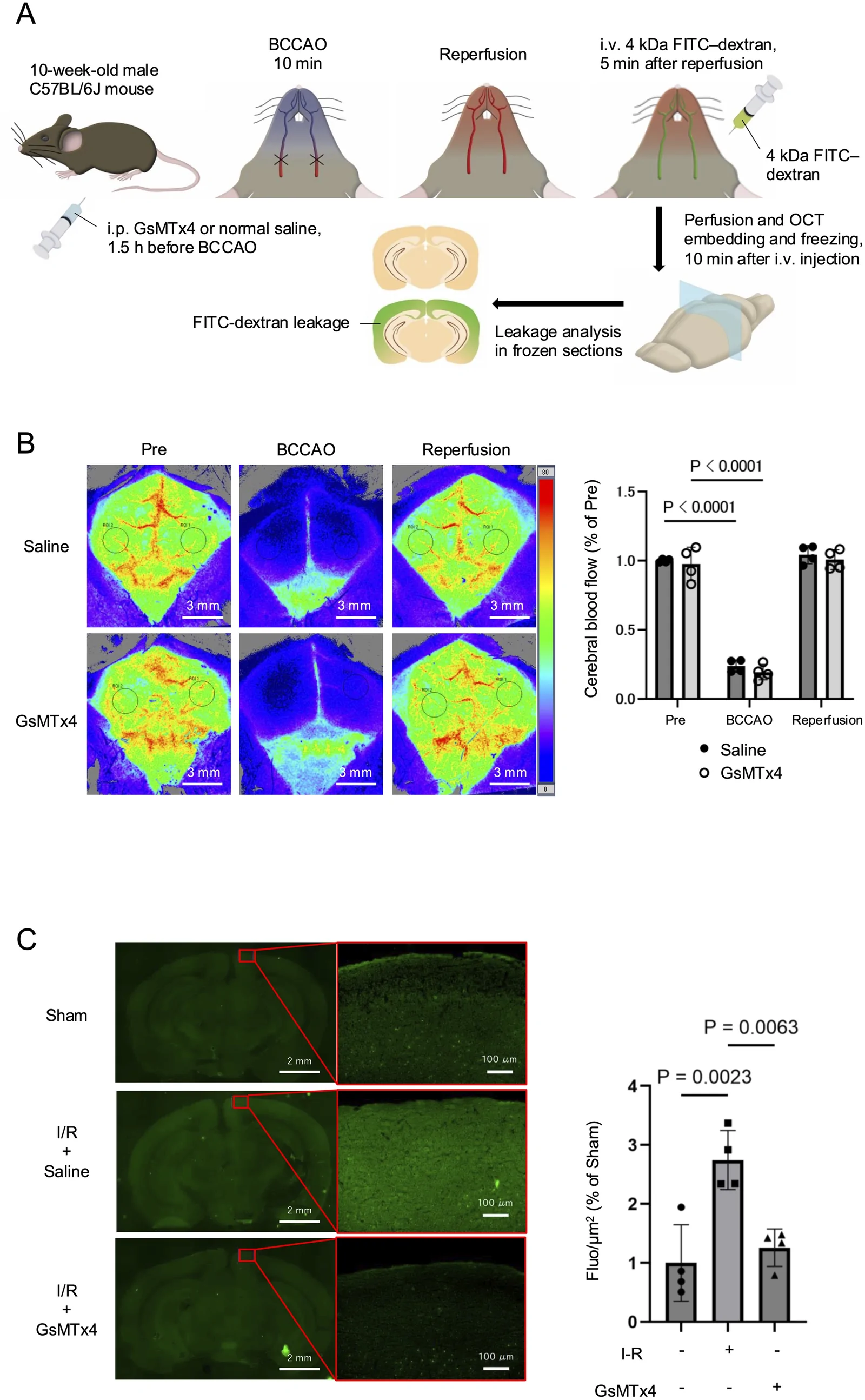
GsMTx4 attenuates vascular permeability after cerebral ischemia-reperfusion. **(A)** A cerebral ischemia-reperfusion (I/R) model was established in 10-week-old male C57BL/6J mice by bilateral common carotid artery occlusion (BCCAO) for 10 min followed by reperfusion. GsMTx4 or saline was administered intraperitoneally 1.5 h before occlusion. Based on the in vitro time course, 4 kDa FITC-dextran was injected intravenously 5 min after reperfusion. Ten minutes later, mice were perfused with heparinized saline, and brains were collected for OCT embedding and cryosectioning. **(B)** Laser speckle contrast imaging showed marked cerebral blood flow reduction during BCCAO and robust recovery after reperfusion, with no significant difference between the I/R + saline and I/R + GsMTx4 groups. Data are mean ± SD (*n* = 4/group). Two-way repeated-measures ANOVA with Geisser-Greenhouse correction: time effect, *P* < 0.0001; treatment effect, *P* = 0.27; interaction, *P* = 0.96. Sidak’s test was used for pairwise comparisons at each time point. Scale bars, 3 mm. **(C)** A 4 kDa FITC-dextran permeability assay showed prominent tracer leakage in the I/R + saline group, which was significantly reduced by GsMTx4. Data are mean ± SD (*n* = 4/group). One-way ANOVA with Tukey’s test: Sham vs. I/R + saline, *P* = 0.0023; Sham vs. I/R + GsMTx4, *P* = 0.76; I/R + saline vs. I/R + GsMTx4, *P* = 0.0063. Scale bars, 2 mm (left) and 100 μm (right)

Given that in vitro tight junction disruption peaked 15–20 min after Piezo1 activation, we evaluated in vivo BBB permeability within the same time window. We also investigated whether GsMTx4 could suppress this ultra-acute permeability increase. Specifically, BCCAO was performed for 10 min, with reperfusion serving as the onset of abrupt hemodynamic stress. Five minutes after reperfusion, 4 kDa FITC–dextran was intravenously administered, followed by a 10-minute circulation period. Brains were then perfused with heparinized saline and rapidly embedded in OCT (Fig. 8A). Three groups were compared: Sham, ischemia/reperfusion (I/R) + saline, and I/R + GsMTx4.

Laser speckle contrast imaging confirmed a marked reduction in CBF during BCCAO and a sharp increase immediately after reperfusion (Fig. 8B), with no difference between I/R + saline and I/R + GsMTx4 groups. Evaluation of FITC–dextran extravasation on the cortical surface revealed significantly increased leakage in the I/R + saline group compared with Sham, indicating BBB disruption in the ultra-acute phase after reperfusion (Fig. 8C). In contrast, this leakage was markedly suppressed in the I/R + GsMTx4 group. These findings are consistent with the involvement of mechanosensitive channel-associated signaling in ultra-acute vascular leakage after reperfusion.

Collectively, these results indicate that vascular leakage increases rapidly after reperfusion and that GsMTx4 attenuates this ultra-acute BBB leakage, supporting a role for mechanosensitive channel-associated signaling in early BBB dysfunction after abrupt hemodynamic stress.

## Discussion

To our knowledge, no study has comprehensively characterized phosphoproteomic changes following Piezo1 activation in brain endothelial cells. In the present study, our findings suggest that pharmacological Piezo1 activation is associated with rapid phosphorylation of the tight junction protein occludin at Tyr287 and endothelial barrier disruption. Pharmacological inhibition further supports the involvement of Src-family kinase-sensitive signaling and is consistent with a contribution of PYK2-associated signaling to these responses. Given that rapid hemodynamic changes critically influence clinical outcomes in cerebrovascular disease, elucidating the role of Piezo1-associated signaling in endothelial responses to flow alteration is pathophysiologically important.

Phosphoproteomic analysis revealed that Yoda1 stimulation significantly enriched GO terms such as cadherin binding, focal adhesion, and actin binding, implicating pathways involved in cell–cell junctions, adhesion, and cytoskeletal dynamics-processes essential for acute barrier regulation. In contrast, enrichment of chromosomal region, histone binding, RNA splicing, and nucleocytoplasmic transport clusters suggested the potential involvement of nuclear and post-transcriptional regulatory processes. Collectively, these enrichment analyses suggest that Piezo1 activation may influence multiple biological processes in brain endothelial cells, including pathways related to endothelial junctional organization and cytoskeletal regulation that are consistent with the observed barrier phenotype. The enrichment of nuclear and post-transcriptional terms may represent additional cellular responses to Piezo1 activation; however, their relevance to BBB function remains uncertain and requires further investigation.

Further enrichment analysis of phosphoproteins increased by Yoda1 and suppressed by the Src inhibitor saracatinib revealed significant alterations in cadherin binding, actin binding, and focal adhesion pathways, suggesting that Piezo1-mediated barrier disruption involves saracatinib-sensitive regulation of junctional and cytoskeletal remodeling. Previous studies in HUVECs and hepatic endothelial cells have reported that Piezo1 activation enhances Src phosphorylation, promoting actin cytoskeletal remodeling and increased vascular permeability [7, 25]. However, the impact of Piezo1 on endothelial barrier function appears to be cell-type dependent. For example, in pulmonary endothelial cells, mechanical stretch–induced Piezo1 activation stabilizes adherens junctions via Ca²⁺-dependent calpain activation and Src inhibition, indicating a protective role [26]. Thus, Piezo1 may exert distinct functions depending on vascular context.

In brain endothelial cells, Piezo1 elicited an exceptionally rapid signaling response. Intracellular Ca²⁺ levels peaked within one minute after Piezo1 stimulation and rapidly declined thereafter (see Additional file 1: Figure **S7****)**. Western blot analysis showed increased phosphorylation of p-Occludin (Tyr287) and p-Src (Tyr416) at one minute, which returned to baseline by five minutes (see Additional file 1: Figure **S8**). Moreover, real-time impedance monitoring using the xCELLigence system revealed that barrier disruption peaked approximately 15 min after stimulation, capturing the ultra-acute dynamic collapse of tight junction integrity. Unlike peripheral endothelium, the cerebral endothelium is constantly exposed to rapid fluctuations in blood flow associated with neuronal activity and blood pressure [27]. Therefore, it must possess mechanisms for immediate mechanosensory adaptation. The observed rapid barrier disruption following Piezo1 activation suggests that Piezo1-associated signaling may participate in the immediate endothelial response to abrupt hemodynamic stress and, under excessive stimulation, contribute to early BBB dysfunction. However, because our experiments relied primarily on pharmacological activation of Piezo1 with Yoda1 rather than direct mechanical stimulation, we did not directly demonstrate that physiological reperfusion-associated mechanical forces activate this signaling pathway. Therefore, the proposed role of Piezo1 signaling in reperfusion-induced mechanotransduction remains to be established.

Early BBB disruption in ischemia–reperfusion injury is known to follow a biphasic pattern, consisting of a mild and transient “early opening” followed by a severe, matrix metallopeptidase-9 (MMP-9)-dependent “late opening” [1]. Shi et al. demonstrated that BBB leakage becomes detectable as early as 30 min after reperfusion, preceding any structural tight-junction breakdown [1]. This early opening was attributed to rapid actin polymerization, junctional protein disassembly (occludin, vascular endothelial-cadherin, ZO-1), and MMP-9–independent mechanisms, whereas the delayed phase at 24–72 h was driven by inflammatory-cell infiltration, MMP-9 release, and basal lamina degradation. Thus, the early phase represents a mechanistically distinct event governed primarily by endothelial cytoskeletal dynamics.

In addition to this early phase, recent studies have also implicated Piezo1 in later-phase BBB disruption. For example, endothelial Piezo1 deletion has been shown to ameliorate BBB breakdown 24 h after reperfusion [28]. Although informative, these mechanisms operate well beyond the ultra-acute window and do not address the initial triggers that precede cytoskeletal remodeling, leukocyte infiltration, and MMP-driven basal lamina degradation. Thus, the molecular signals that initiate BBB failure within minutes after the restoration of blood flow have remained essentially unknown.

Our observation that Piezo1 activation induces ultra-acute barrier leakage within 10–20 min—well before the earliest time points examined by Shi et al. [1].—aligns with this two-phase framework and suggests that Piezo1-associated, saracatinib-sensitive signaling may contribute to the earliest underlying events early BBB opening. Notably, inhibition of this pathway markedly attenuated tight-junction disassembly and preserved barrier function, raising the possibility that Piezo1-dependent mechanotransduction may contribute to subsequent barrier deterioration and later inflammatory injury. However, whether modulation of this pathway influences long-term BBB dysfunction or neurological outcomes remains to be determined.

This concept may have potential clinical relevance. Several clinical studies have demonstrated that contrast extravasation immediately after mechanical thrombectomy strongly predicts hemorrhagic transformation, poor functional outcome, and mortality [3, 29]. These clinical observations are conceptually consistent with the possibility that ultra-acute endothelial responses to rapid hemodynamic restoration may influence downstream pathology. However, the present study did not employ a thrombectomy model or assess hemorrhagic transformation, long-term BBB integrity, or neurological outcomes. Therefore, the potential clinical relevance of Piezo1-associated signaling remains speculative and warrants further investigation in clinically relevant experimental models.

Our pharmacological experiments suggest that Yoda1-associated occludin Tyr287 phosphorylation involves Src-associated signaling and may preferentially involve PYK2 rather than FAK. Defactinib attenuated occludin phosphorylation and barrier dysfunction, supporting the involvement of defactinib-sensitive signaling downstream of Src. Notably, several studies have linked occludin phosphorylation to endothelial barrier regulation [30]. At Tyr287, prior work has suggested a barrier-protective role in certain contexts [19, 31]. In contrast, in human brain endothelial cells we observed acute Piezo1 activation to increase Tyr287 phosphorylation and weaken the barrier, indicating that Tyr287 is context-dependent. Therefore, the present findings should be interpreted as demonstrating an association between Tyr287 phosphorylation and barrier dysfunction rather than establishing Tyr287 phosphorylation as a direct causal mediator of BBB disruption. Determining whether Tyr287 phosphorylation is necessary or sufficient for Yoda1-induced barrier dysfunction will require site-directed mutational analysis of Tyr287. Moreover, other occludin phosphorylation sites may also contribute to the observed barrier phenotype. Occludin phosphorylation is known to regulate endothelial barrier function in a site- and context-dependent manner. In particular, Ser490 phosphorylation has been implicated in VEGF-induced endothelial permeability and angiogenesis [10]. In our experimental system, VEGF did not induce Tyr287 phosphorylation (see Additional file 1: Figure **S9**), indicating that Tyr287 phosphorylation is not invariably induced by a permeability-promoting stimulus. However, this finding does not establish that Tyr287 phosphorylation is specific to Piezo1 activation or that Yoda1 and VEGF act through mutually exclusive pathways. Because Ser490 phosphorylation was not assessed in the present study, its potential contribution to Yoda1-responsive barrier dysfunction cannot be excluded. Further studies using validated site-specific reagents or complementary molecular approaches will be required to define the respective roles of Tyr287, Ser490, and other occludin phosphorylation sites.

As limitations, our in vitro experiments employed a chemical agonist rather than direct shear stress stimulation. Although GsMTx4 attenuated ultra-acute BBB leakage in the BCCAO reperfusion model, this pharmacological observation should be interpreted as evidence supporting the involvement of mechanosensitive channel-associated signaling rather than direct evidence of endothelial Piezo1 activation in vivo. Moreover, we did not examine Src activation, PYK2 activation, or occludin Tyr287 phosphorylation in vivo. Therefore, the complete Piezo1–Src–PYK2–occludin signaling pathway proposed from our in vitro studies remains to be validated using endothelial-specific genetic and molecular approaches.

A second limitation is that our in vitro experiments were performed using the immortalized human brain endothelial cell line HBEC-5i. Although HBEC-5i cells are widely used as a model of human brain endothelium, they do not fully recapitulate the highly restrictive phenotype and multicellular interactions of the BBB in vivo. Therefore, the present findings should be interpreted with caution, and future studies using primary human brain microvascular endothelial cells, induced pluripotent stem cell-derived BBB models, or multicellular neurovascular unit systems incorporating astrocytes and pericytes will be important to further establish the physiological relevance of the Piezo1-associated Src/PYK2 signaling.

A further limitation, our study focused exclusively on the ultra-acute phase of BBB disruption, and we did not assess later-phase mechanisms such as leukocyte infiltration or MMP-mediated basal lamina degradation, which characterize inflammatory BBB breakdown. Finally, although tight junction integrity represents a central component of BBB function, we did not examine other critical barrier mechanisms such as vesicular transcytosis or efflux transporter activity. Future studies are required to determine how Piezo1 signaling influences these additional BBB regulatory pathways.

## Conclusions

This study identifies rapid phosphorylation changes associated with intercellular junctions, adhesion, and cytoskeletal dynamics following pharmacological Piezo1 activation in brain endothelial cells. The findings support the involvement of Src/PYK2-associated signaling in Yoda1-induced barrier dysfunction and suggest that mechanosensitive channel-associated signaling contributes to ultra-acute BBB leakage after reperfusion. Further studies are required to establish the specific molecular pathway and its therapeutic relevance.

## Supplementary Information

Below is the link to the electronic supplementary material.

Supplementary Material 1: Additional file 1: File format: PDF: Title of data: Supplementary Figures 1–9. Description of data: Supplementary figures and figure legends showing additional experimental results supporting the main findings.

Supplementary Material 2: Additional file 2: File format: DOCX: Title of data: Supplementary Methods. Description of data: Detailed experimental procedures, cell proliferation assay (CCK-8), intracellular Ca²⁺ imaging and live-cell imaging.

Supplementary Material 3: Additional file 3: File format: XLSX: Title of data: Supplementary Table 1. Gene Ontology (GO) enrichment analysis of phosphoproteins upregulated by Yoda1 stimulation. Description of data: This table lists the significantly enriched Gene Ontology (GO) terms identified from phosphoproteins whose phosphorylation levels were significantly increased in HBEC-5i cells following Yoda1 stimulation compared with control (CTRL).

Supplementary Material 4: Additional file 4: File format: XLSX: Title of data: Supplementary Table 2. KEGG pathway enrichment analysis of phosphoproteins upregulated by Yoda1 stimulation. Description of data: This table summarizes the significantly enriched KEGG pathways identified from phosphoproteins whose phosphorylation levels were significantly increased in HBEC-5i cells following Yoda1 stimulation compared with control (CTRL).

Supplementary Material 5: Additional file 5: File format: XLSX: Title of data: Supplementary Table 3. Reactome pathway enrichment analysis of phosphoproteins upregulated by Yoda1 stimulation. Description of data: This table presents the significantly enriched Reactome pathways identified from phosphoproteins whose phosphorylation levels were significantly increased in HBEC-5i cells following Yoda1 stimulation compared with control (CTRL).

Supplementary Material 6: Additional file 6: File format: XLSX: Title of data: Supplementary Table 4. Gene Ontology (GO) enrichment analysis of phosphoproteins downregulated by Yoda1 stimulation. Description of data: This table lists the significantly enriched Gene Ontology (GO) terms identified from phosphoproteins whose phosphorylation levels were significantly decreased in HBEC-5i cells following Yoda1 stimulation compared with control (CTRL).

Supplementary Material 7: Additional file 7: File format: XLSX: Title of data: Supplementary Table 5. Gene Ontology (GO) enrichment analysis of saracatinib-sensitive phosphoproteins. Description of data: This table lists the significantly enriched Gene Ontology (GO) terms identified from phosphoproteins whose phosphorylation was significantly increased by Yoda1 stimulation but was attenuated by co-treatment with the Src inhibitor saracatinib.

Supplementary Material 8: Additional file 8: File format: XLSX: Title of data: Supplementary Table 6. Reactome pathway enrichment analysis of saracatinib-sensitive phosphoproteins. Description of data: This table presents the significantly enriched Reactome pathways identified from phosphoproteins whose phosphorylation was significantly increased by Yoda1 stimulation but was attenuated by co-treatment with the Src inhibitor saracatinib.

Supplementary Material 9: Additional file 9: File format: MP4: Title of data: Supplementary Movie 1. Live-cell imaging of HBEC-5i under control conditions. Description of data: Cells maintained stable morphology and tight intercellular junctions throughout the 40-min recording.

Supplementary Material 10: Additional file 10: File format: MP4: Title of data: Supplementary Movie 2. Live-cell imaging of HBEC-5i stimulated with Yoda1 (15 µM). Description of data: Upon Piezo1 activation by Yoda1 (15 µM), HBEC-5i cells exhibited dynamic morphological changes characterized by transient cell contraction and intercellular gap formation within minutes after stimulation.

Supplementary Material 11: Additional file 11: File format: MP4: Title of data: Supplementary Movie 3. Live-cell imaging of HBEC-5i treated with Yoda1 (15 µM) and saracatinib (5 µM). Description of data: Co-treatment with saracatinib (5 µM) attenuated Yoda1-responsive cell retraction and gap formation, maintaining more continuous cell–cell borders compared to Yoda1 alone.

Supplementary Material 12. File format: JPG. Title of data: Graphical Abstract. Description of data: A graphical summary of the study and its key findings.

Supplementary Material 13. File format: PDF. Title of data: Uncropped Western blot images. Description of data: Uncropped Western blot images of the membrane strips used for Figures 3A, 6A, and 7A and Supplementary Figures S4, S8, and S9.

## Data Availability

The datasets used and/or analysed during the current study are available from the corresponding author on reasonable request.

## Abbreviations

BBB: Blood-brain barrier
BCCAO: Bilateral common carotid artery occlusion
CBF: Cerebral blood flow
CCK-8: Cell Counting Kit-8
CI: Cell Index
CTRL: Control
FAK: Focal adhesion kinase
FDR: False discovery rate
FITC: Fluorescein isothiocyanate
F-actin: Filamentous actin
GO: Gene Ontology
HBEC-5i: Human brain microvascular endothelial cell line-5i
HBECs: Human brain microvascular endothelial cells
I/R: Ischemia/reperfusion
KEGG: Kyoto Encyclopedia of Genes and Genomes
LC-MS/MS: Liquid chromatography-tandem mass spectrometry
MMP-9: Matrix metallopeptidase-9
OCT: Optimal cutting temperature compound
PCA: Principal component analysis
PYK2: Proline-rich tyrosine kinase 2
RM-ANOVA: Repeated-measures analysis of variance
SFKs: Src family kinases
TiO₂: Titanium dioxide
VEGF: Vascular endothelial growth factor
ZO-1: Zonula occludens-1
