# Supplementary material for: Piezo1–Src signaling rapidly disrupts brain endothelial barrier integrity
Source: Fluids Barriers CNS. 2026 Aug 13;23:112. doi: 10.1186/s12987-026-00859-6 (PMC13613767; doi:10.1186/s12987-026-00859-6)

Supplementary file:
Uncropped western blot images of membrane strips used for Figures 3A, 6A, 7A, and Supplementary Figures S4, S8, and S9

Red boxes indicate the regions shown in the main figures. Molecular weight markers are indicated on the right.

Figure 3A

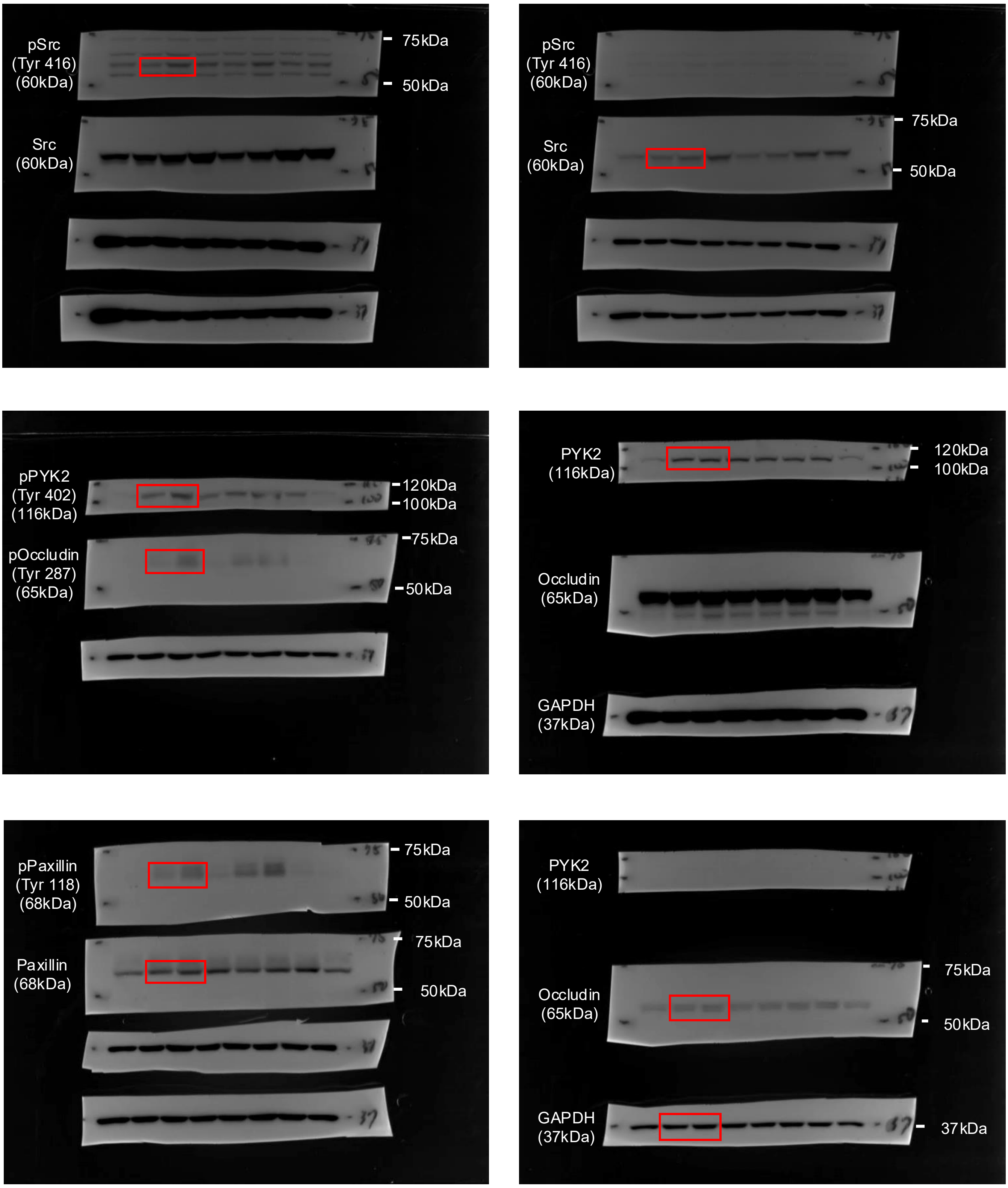

Figure 6A

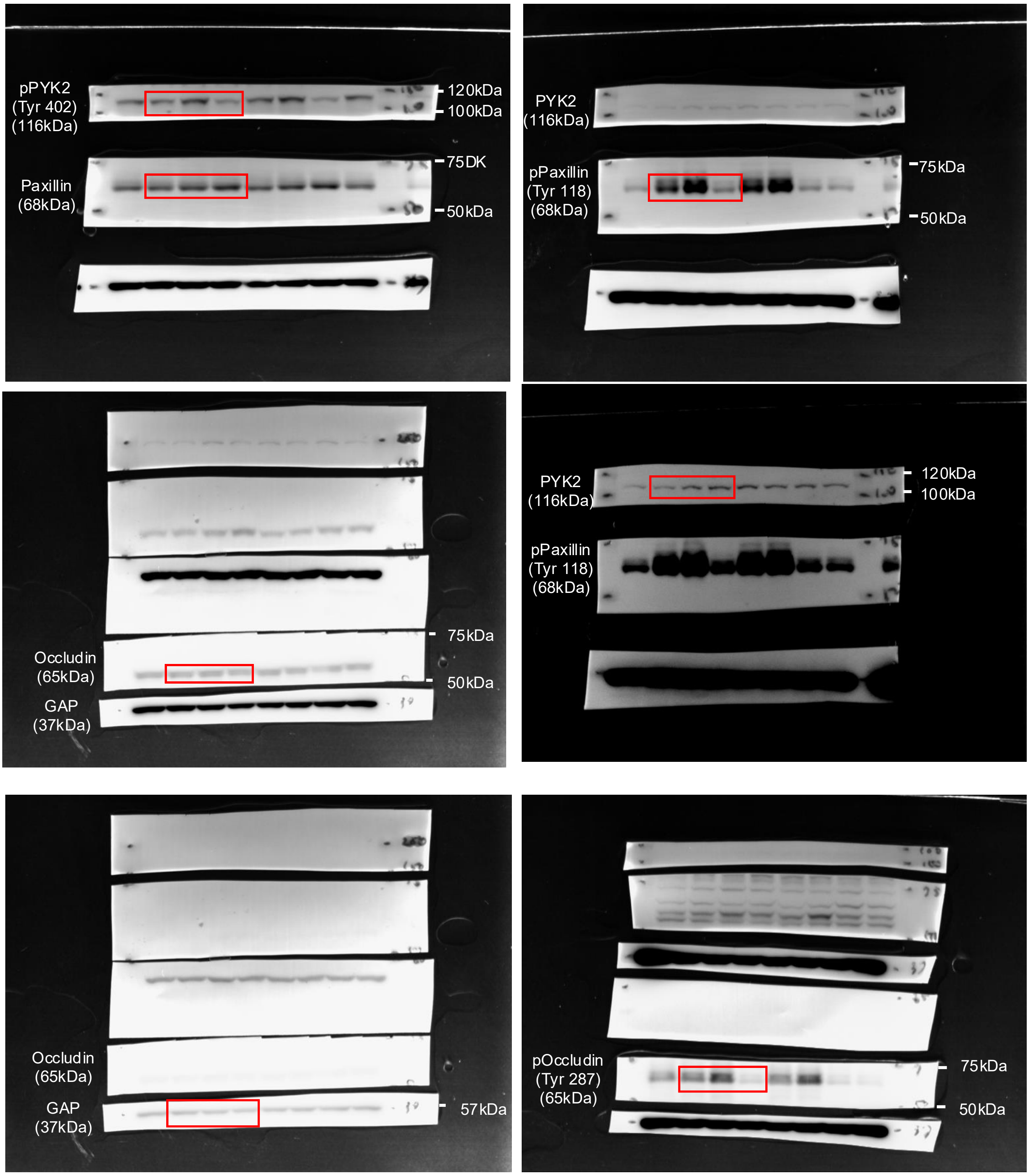
pPYK2
(Tyr 402)
(116kDa)
120kDa
100kDa
Paxillin
(68kDa)
75DK
50kDa
PYK2
(116kDa)
pPaxillin
(Tyr 118)
(68kDa)
75kDa
50kDa
75kDa
Occludin
(65kDa)
50kDa
GAP
(37kDa)
PYK2
(116kDa)
120kDa
100kDa
pPaxillin
(Tyr 118)
(68kDa)
Occludin
(65kDa)
GAP
(37kDa)
57kDa
pOccludin
(Tyr 287)
(65kDa)
75kDa
50kDa

Figure 7A

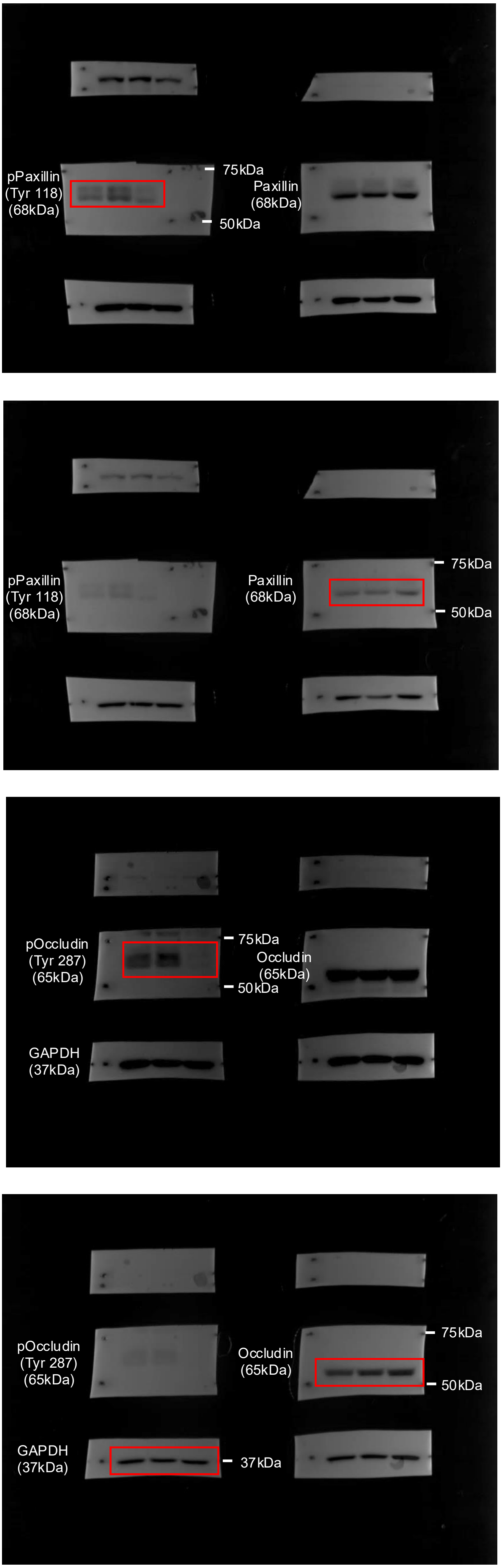

pPaxillin
(Tyr 118)
(68kDa)
75kDa
50kDa
Paxillin
(68kDa)
pPaxillin
(Tyr 118)
(68kDa)
Paxillin
(68kDa)
75kDa
50kDa
pOccludin
(Tyr 287)
(65kDa)
75kDa
50kDa
Occludin
(65kDa)
GAPDH
(37kDa)
pOccludin
(Tyr 287)
(65kDa)
Occludin
(65kDa)
75kDa
50kDa
GAPDH
(37kDa)
37kDa

Supplementary Figure 4A

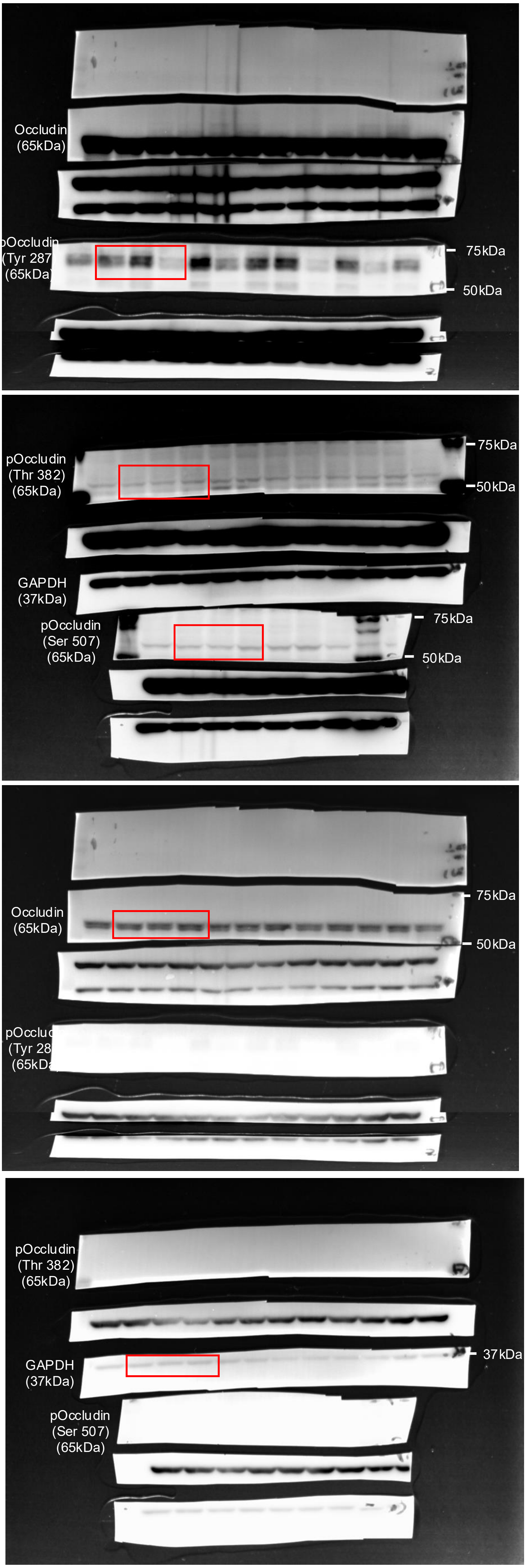

Supplementary Figure 4B

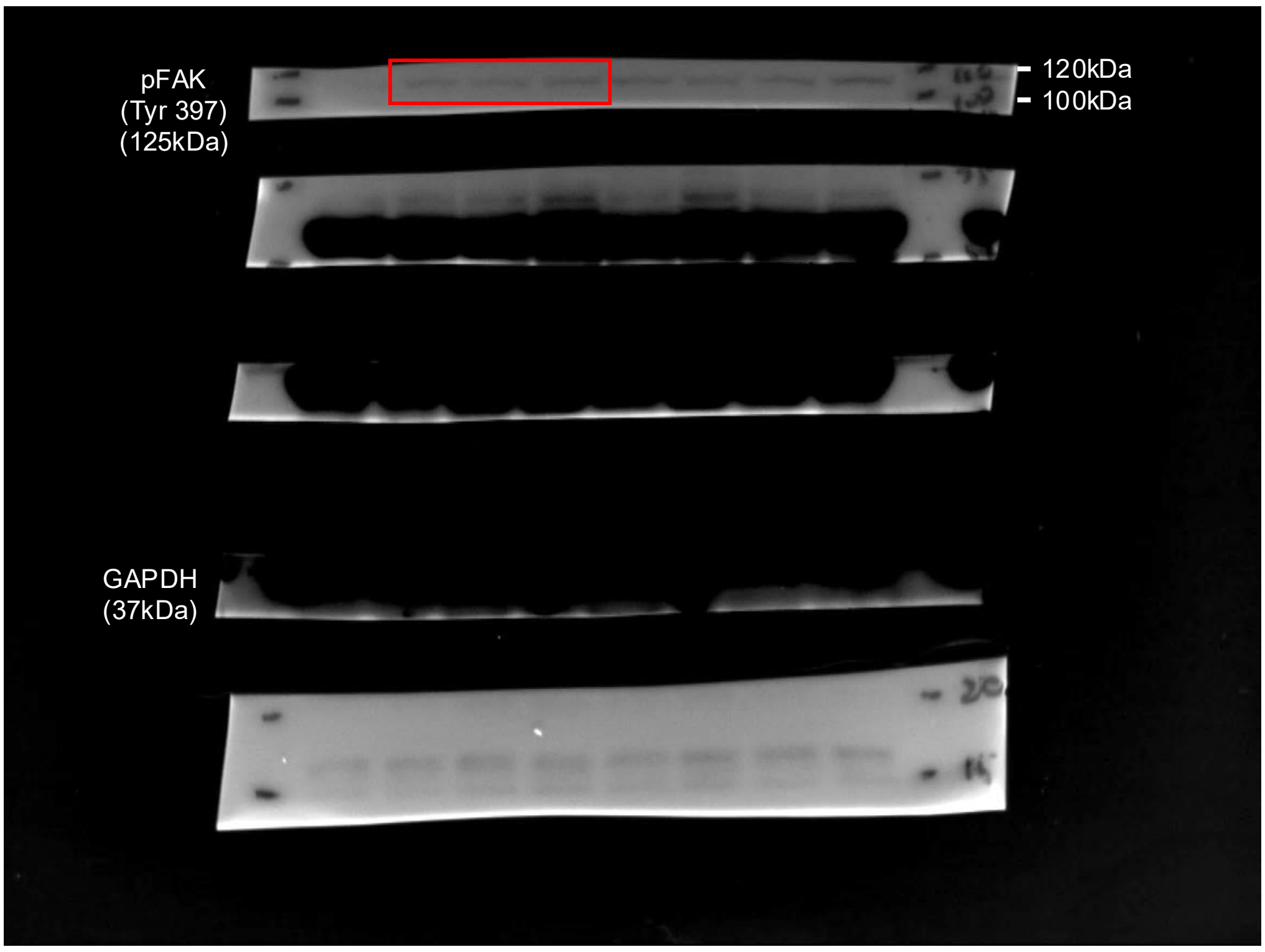

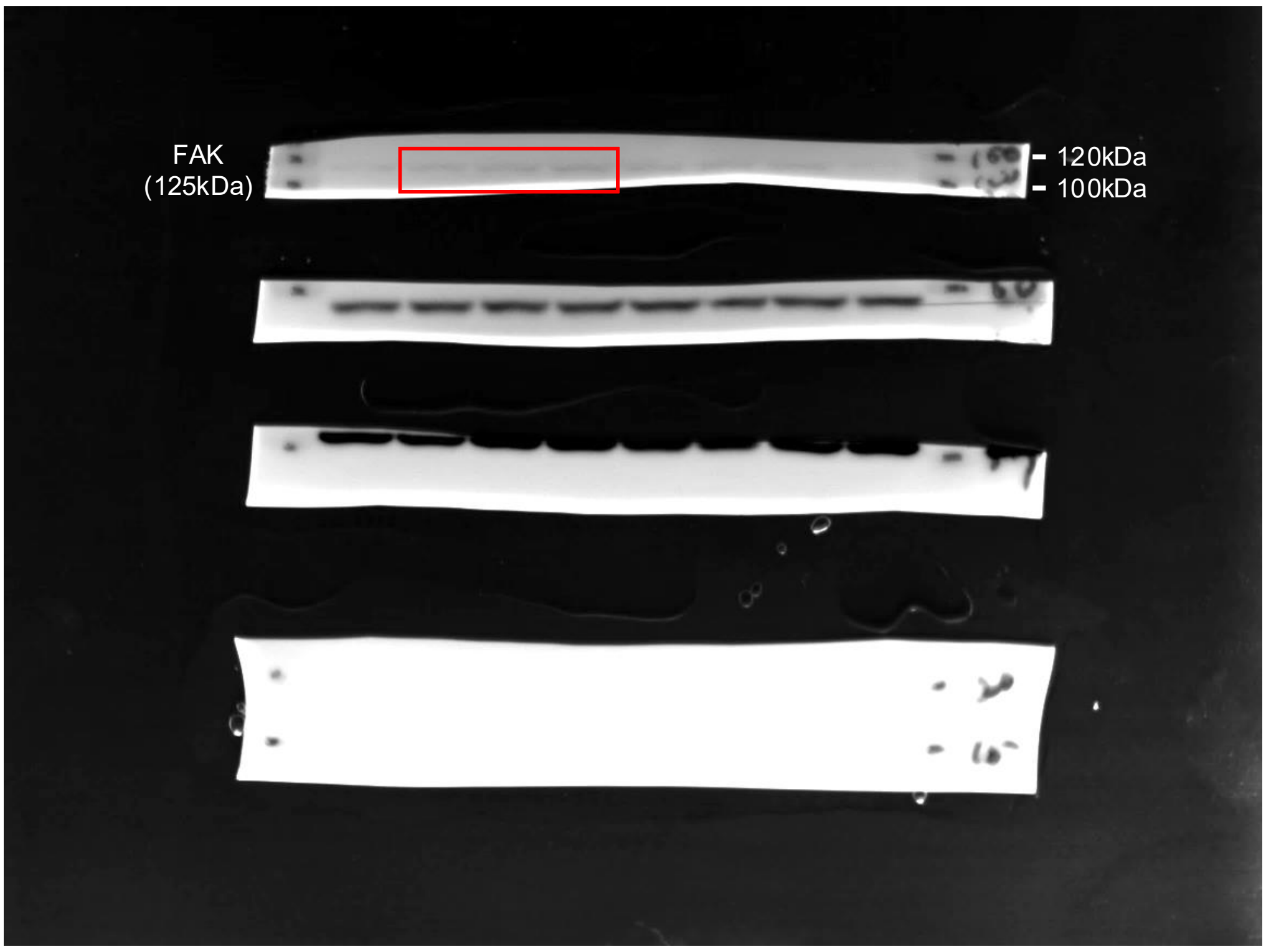

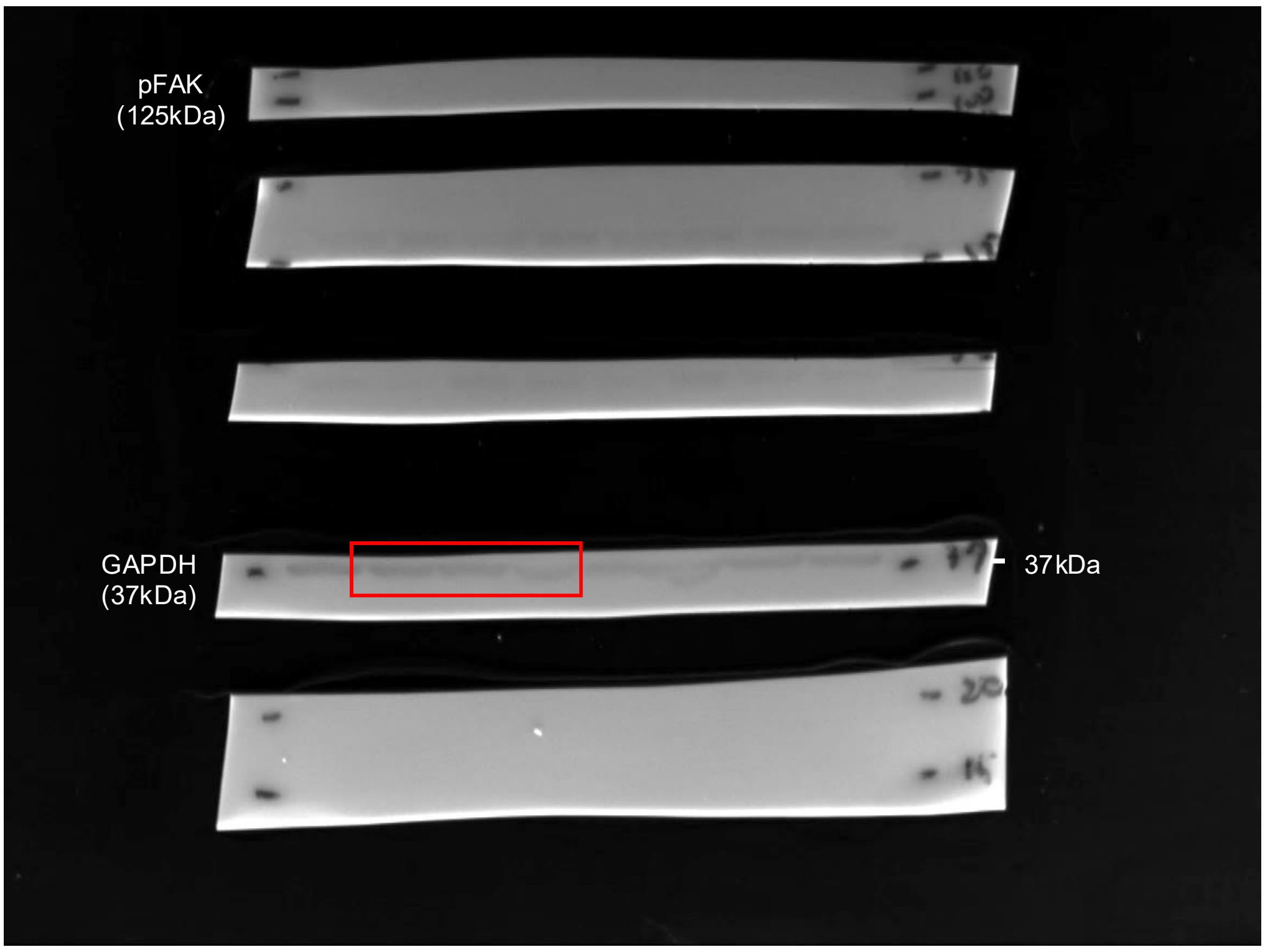

Supplementary Figure 8

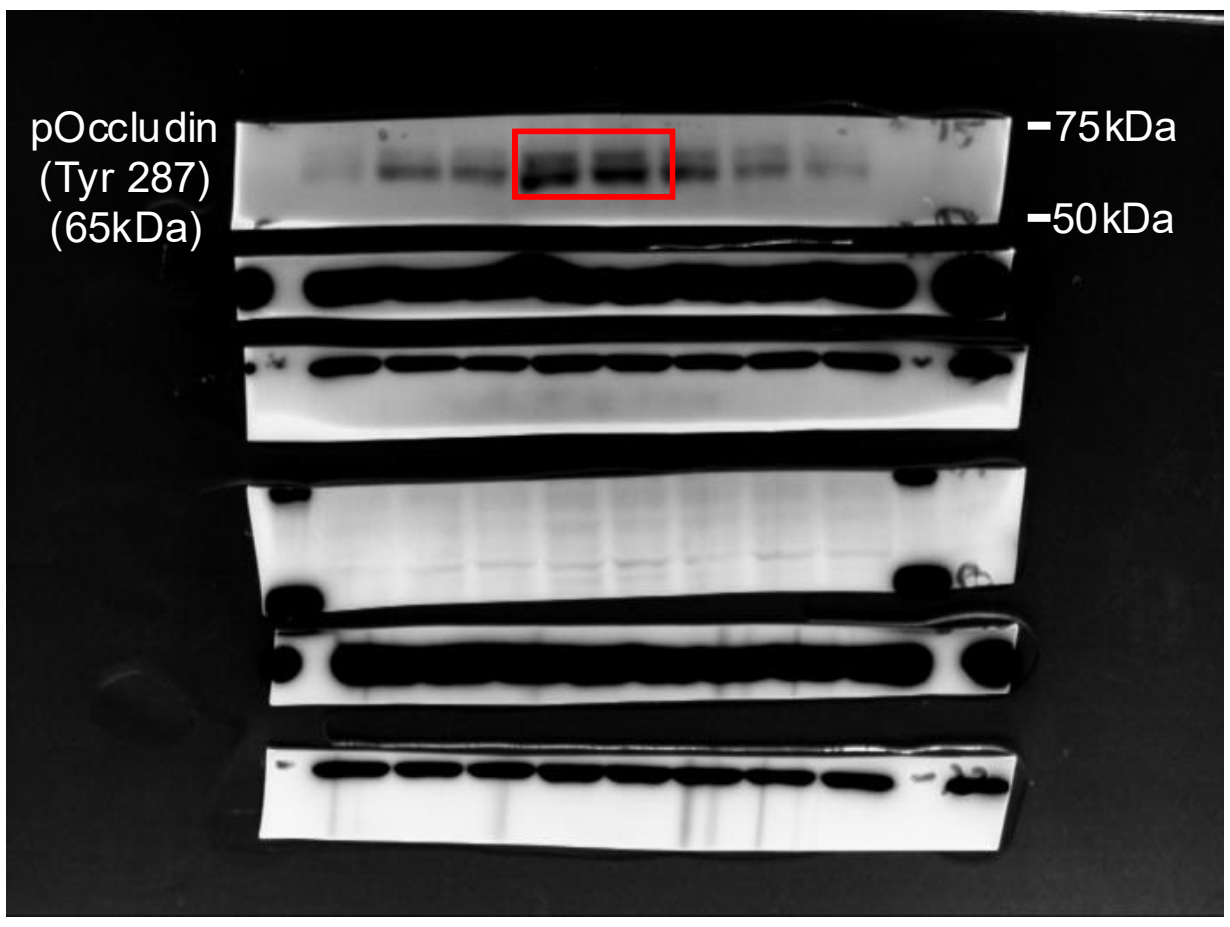

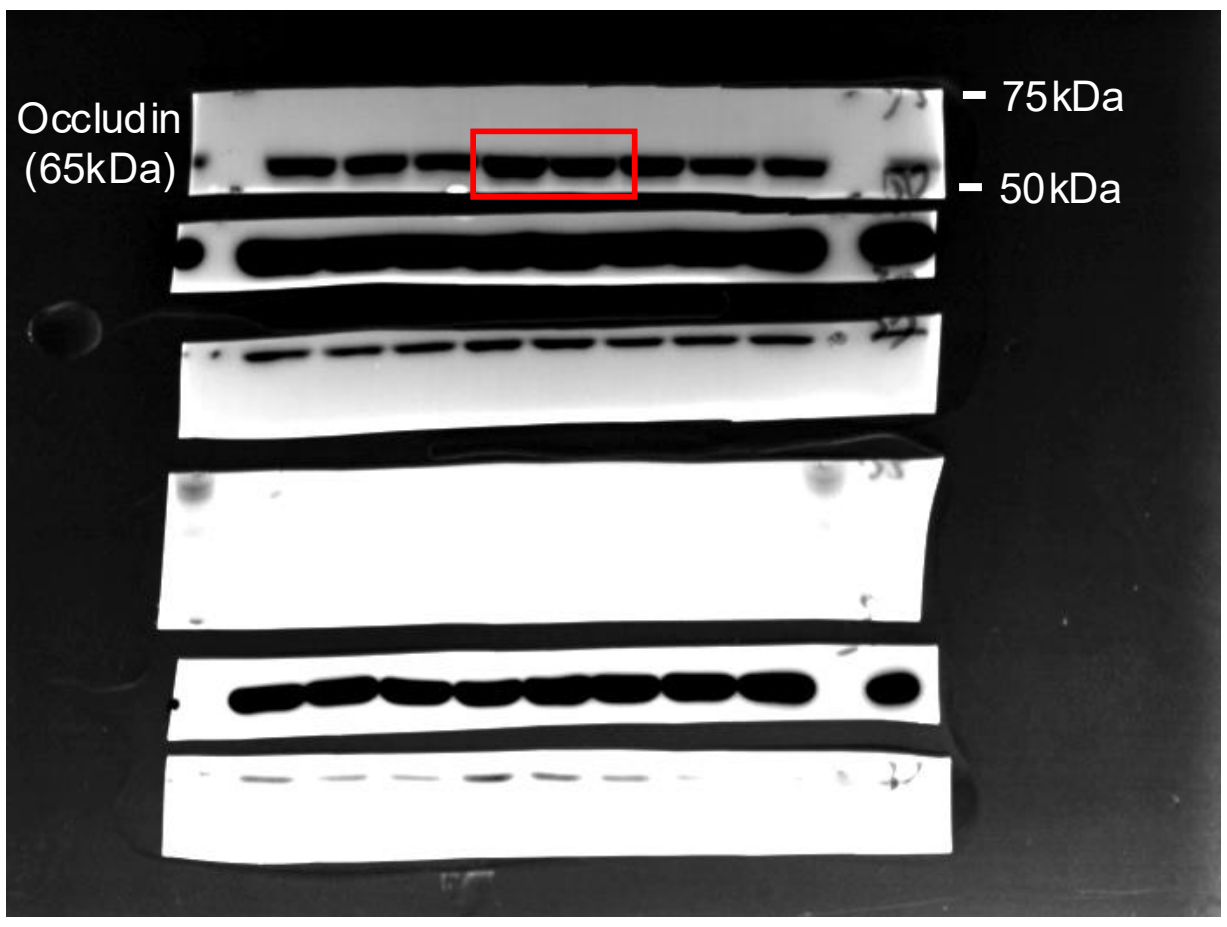

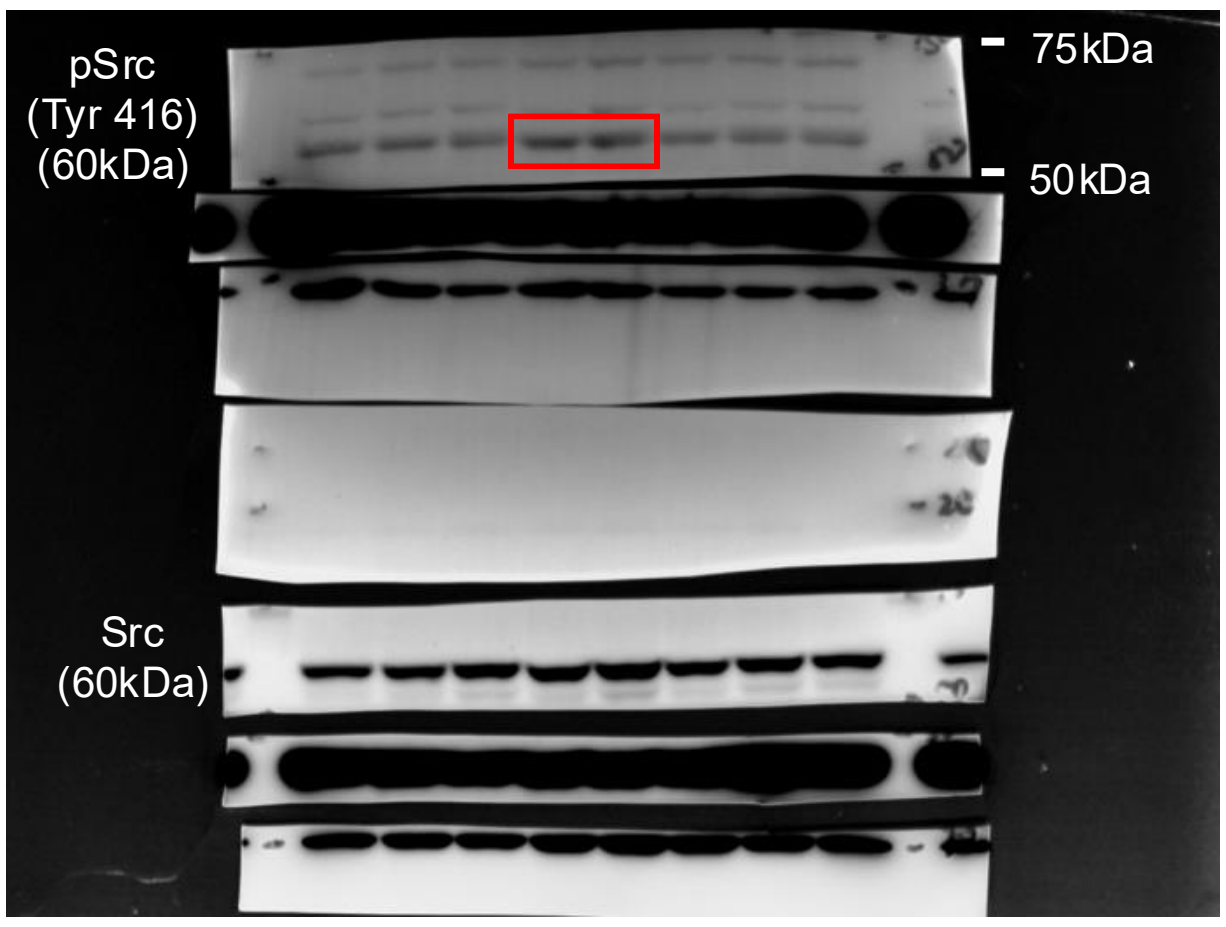

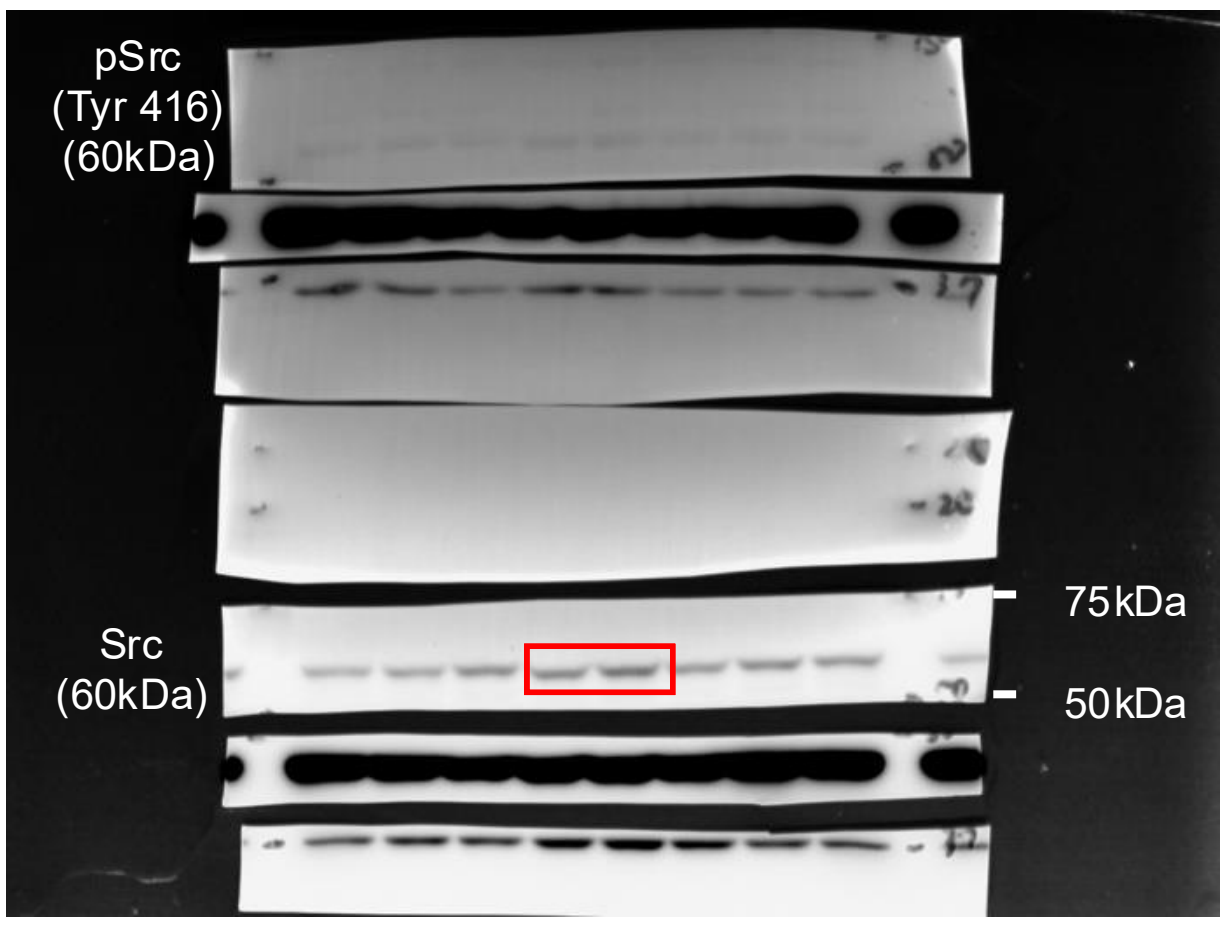

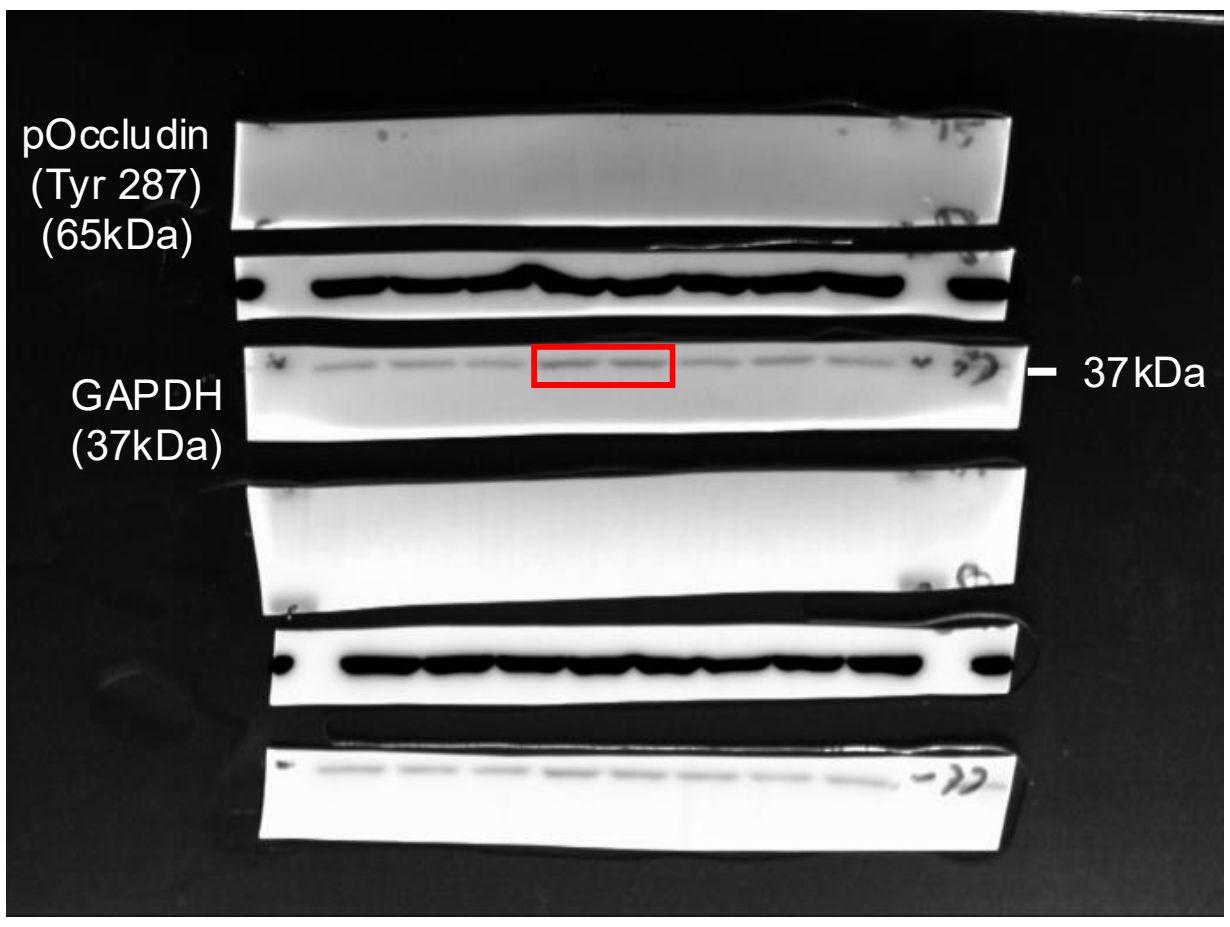

Supplementary Figure 9

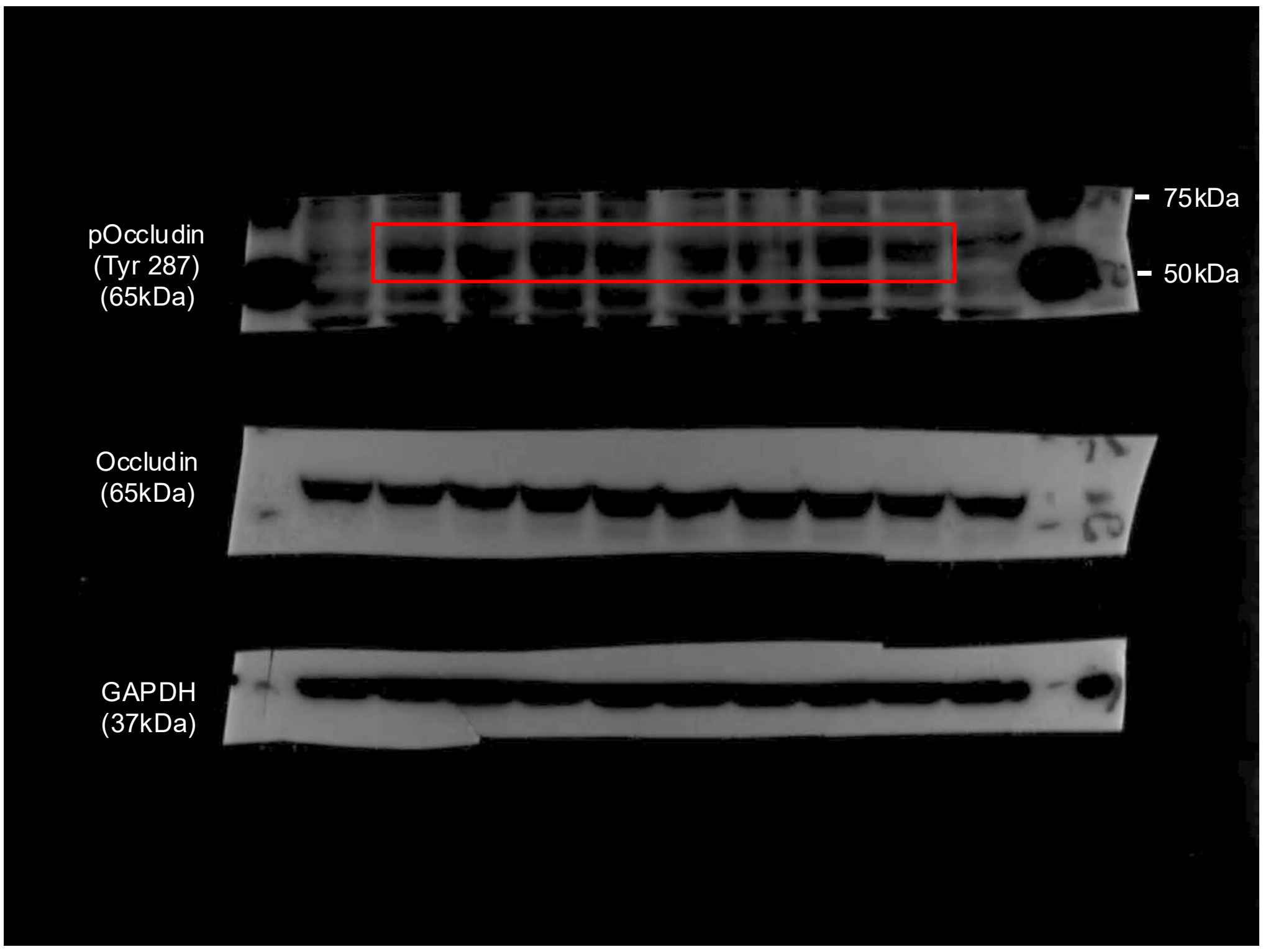

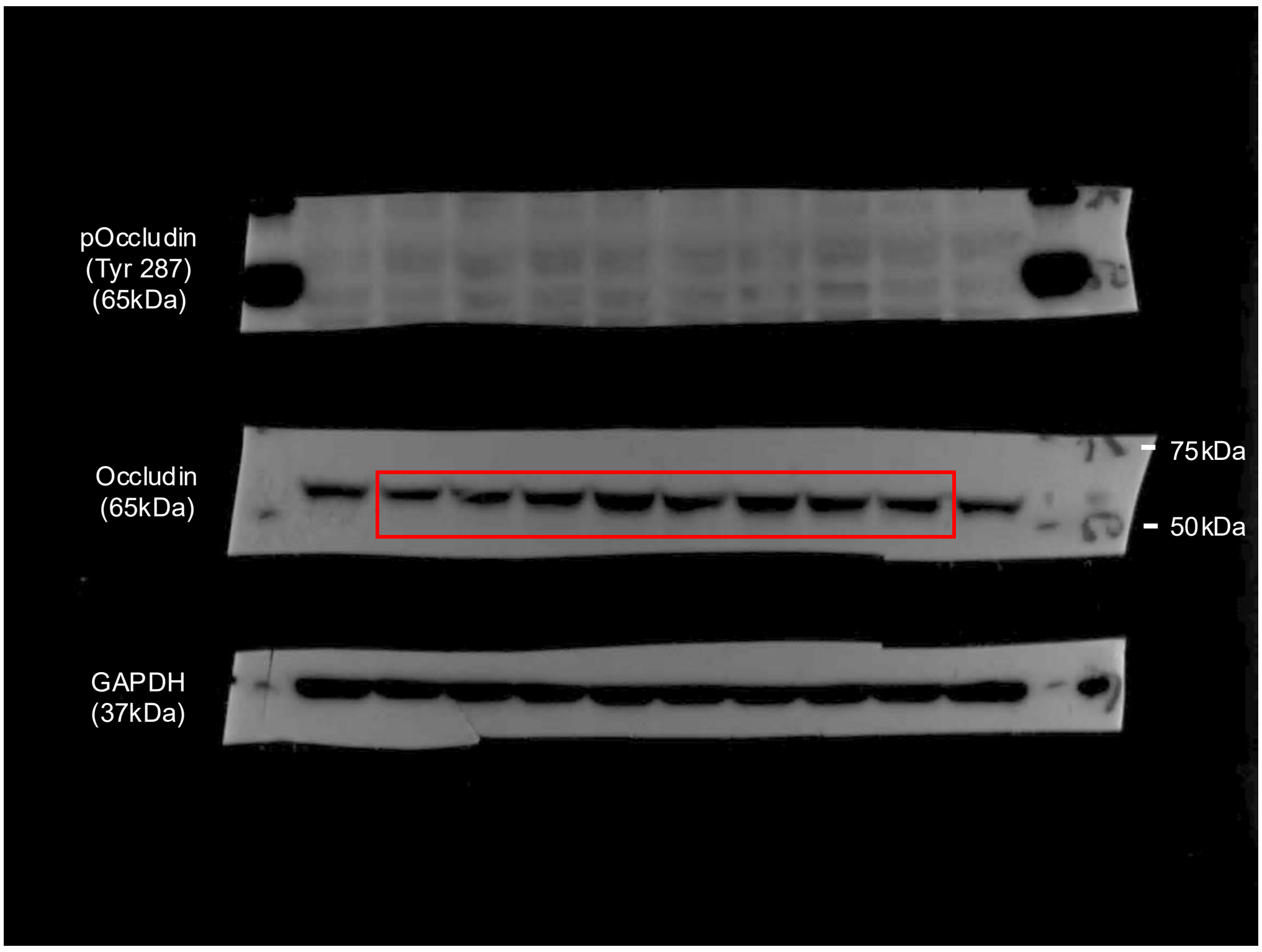

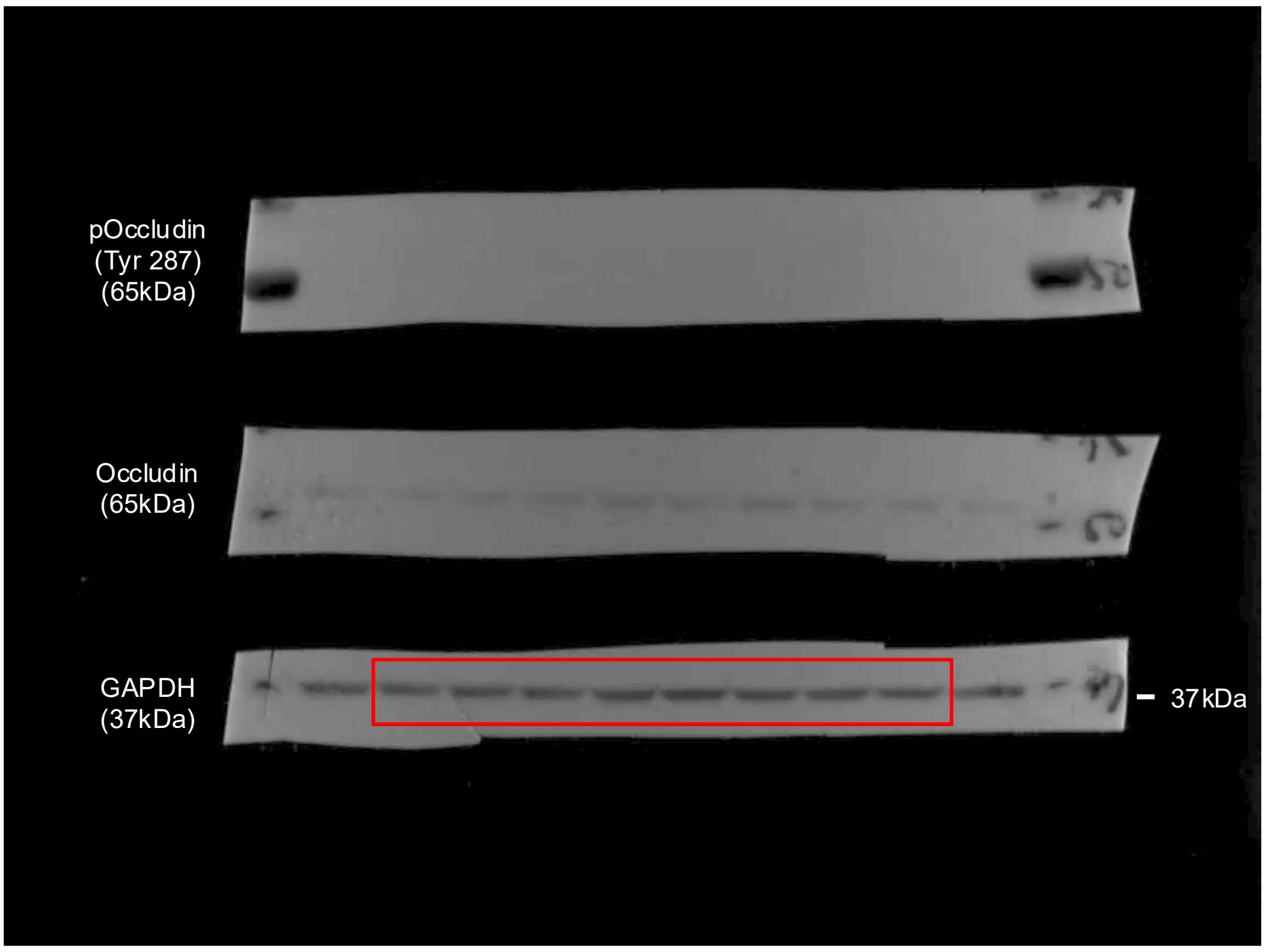

Supplement: Supplementary file 1 [file 12987_2026_859_MOESM13_ESM.pdf]
