# Supplementary material for: Piezo1–Src signaling rapidly disrupts brain endothelial barrier integrity
Source: Fluids Barriers CNS. 2026 Aug 13;23:112. doi: 10.1186/s12987-026-00859-6 (PMC13613767; doi:10.1186/s12987-026-00859-6)
Supplement: Supplementary file 2 [file 12987_2026_859_MOESM1_ESM.pdf]

Supplementary Figures

Supplementary Figure 1

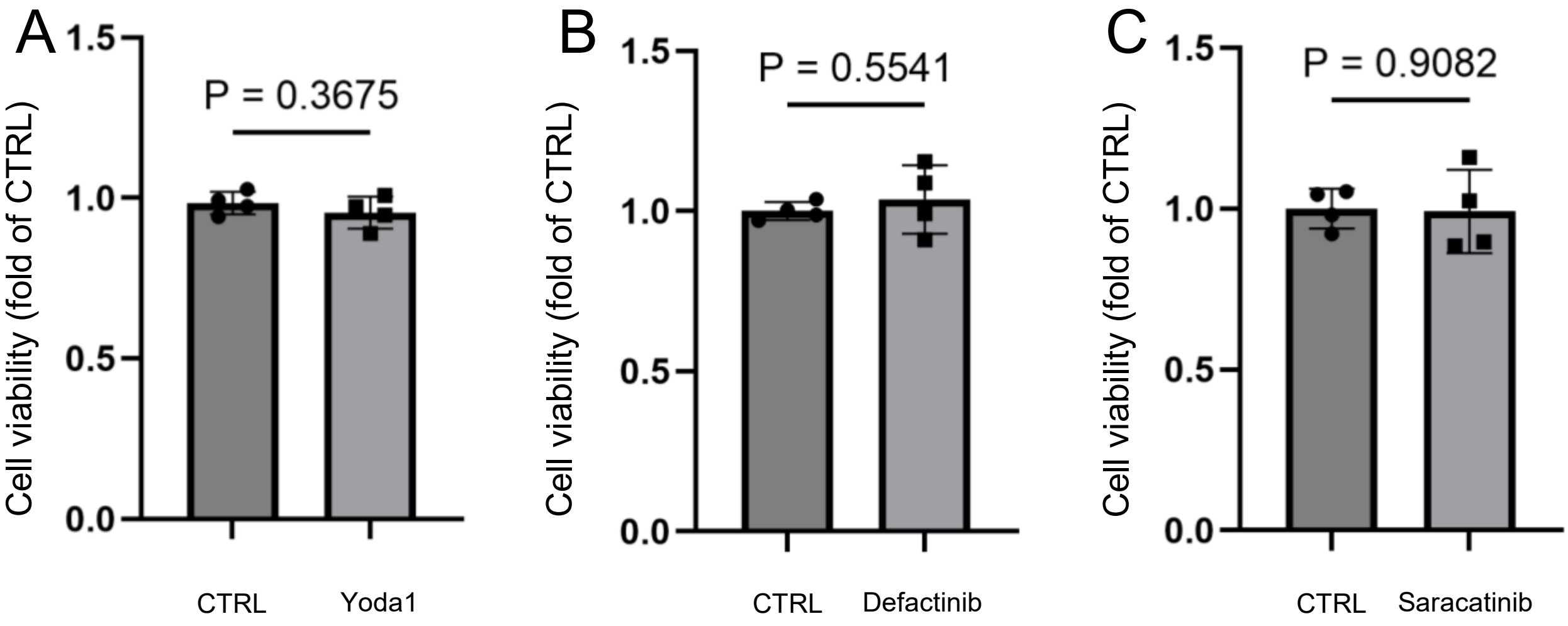

Supplementary Figure 1. Cell viability screening for drug concentration selection.

Cell viability was assessed by CCK-8 assay after 24 h exposure to each compound in human brain microvascular endothelial cells (HBEC-5i). (A) Yoda1, (B) defactinib, and (C) saracatinib at the indicated concentrations. Absorbance values were normalised to control (CTRL). Data are presented as mean ± SD (n = 4). Group differences were analysed by one-way ANOVA followed by Tukey's post hoc test; no statistically significant differences were detected compared with CTRL and each compound at the tested concentration ($P > 0.05$ for all comparisons). Final working concentrations were selected based on these CCK-8 viability data to avoid effects on proliferation/viability: (A) Yoda1 15 μM, (B) defactinib 2 μM, and (C) saracatinib 5 μM. (ns, not significant)

Supplementary Figure 2

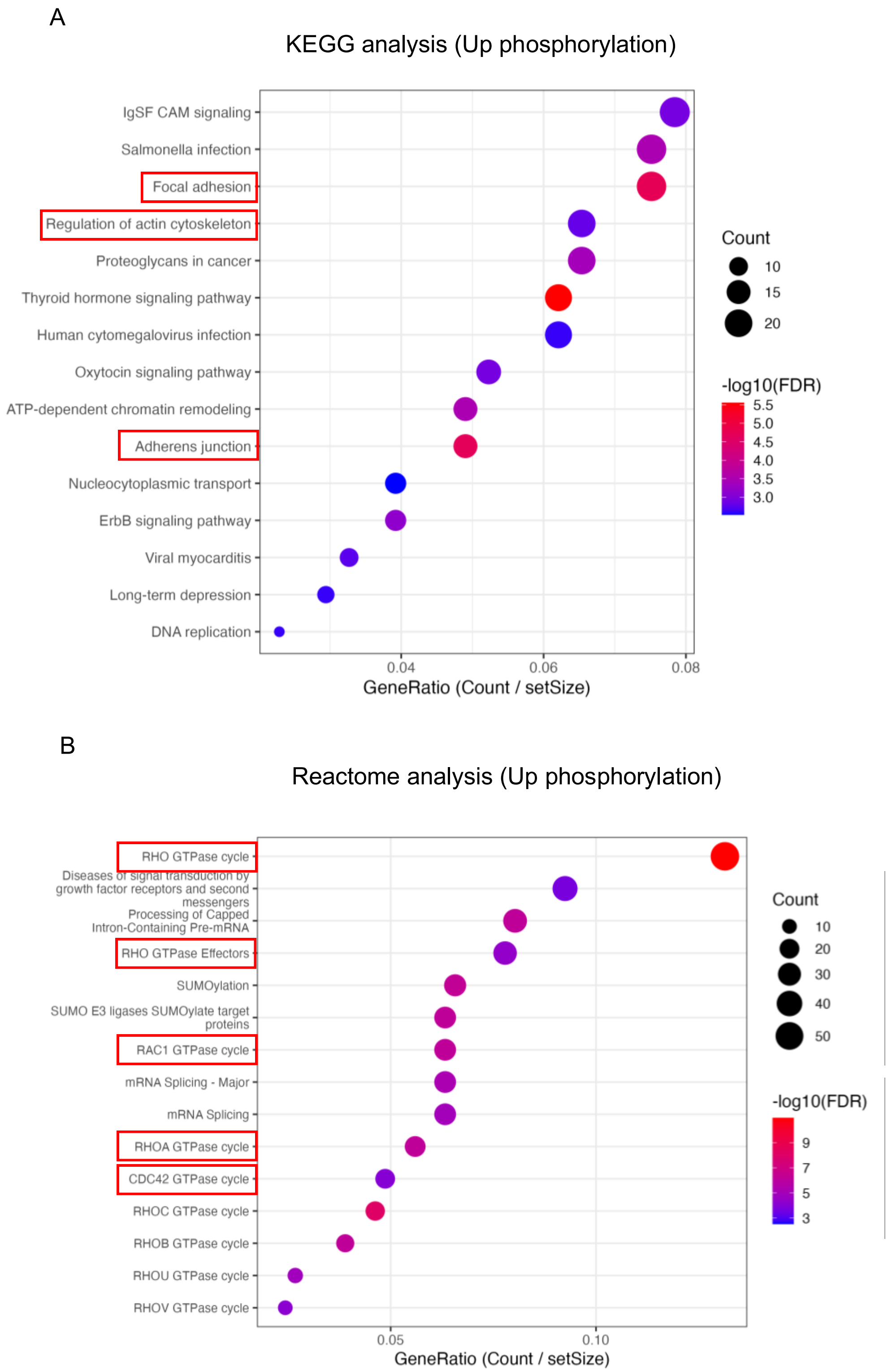

Supplementary Figure 2. KEGG and Reactome enrichment of phosphoproteins upregulated by Yoda1.
Dot plots show the top 15 enriched pathways for phosphoproteins whose phosphorylation increased after Yoda1 stimulation: (A) KEGG and (B) Reactome. The x-axis indicates GeneRatio (Count/setSize); dot size denotes the number of mapped proteins (Count); dot color represents –log10(FDR). Red boxes highlight junction/cytoskeleton-related terms (e.g., focal adhesion, adherens junction, RHO GTPase cycle).

Supplementary Figure 3

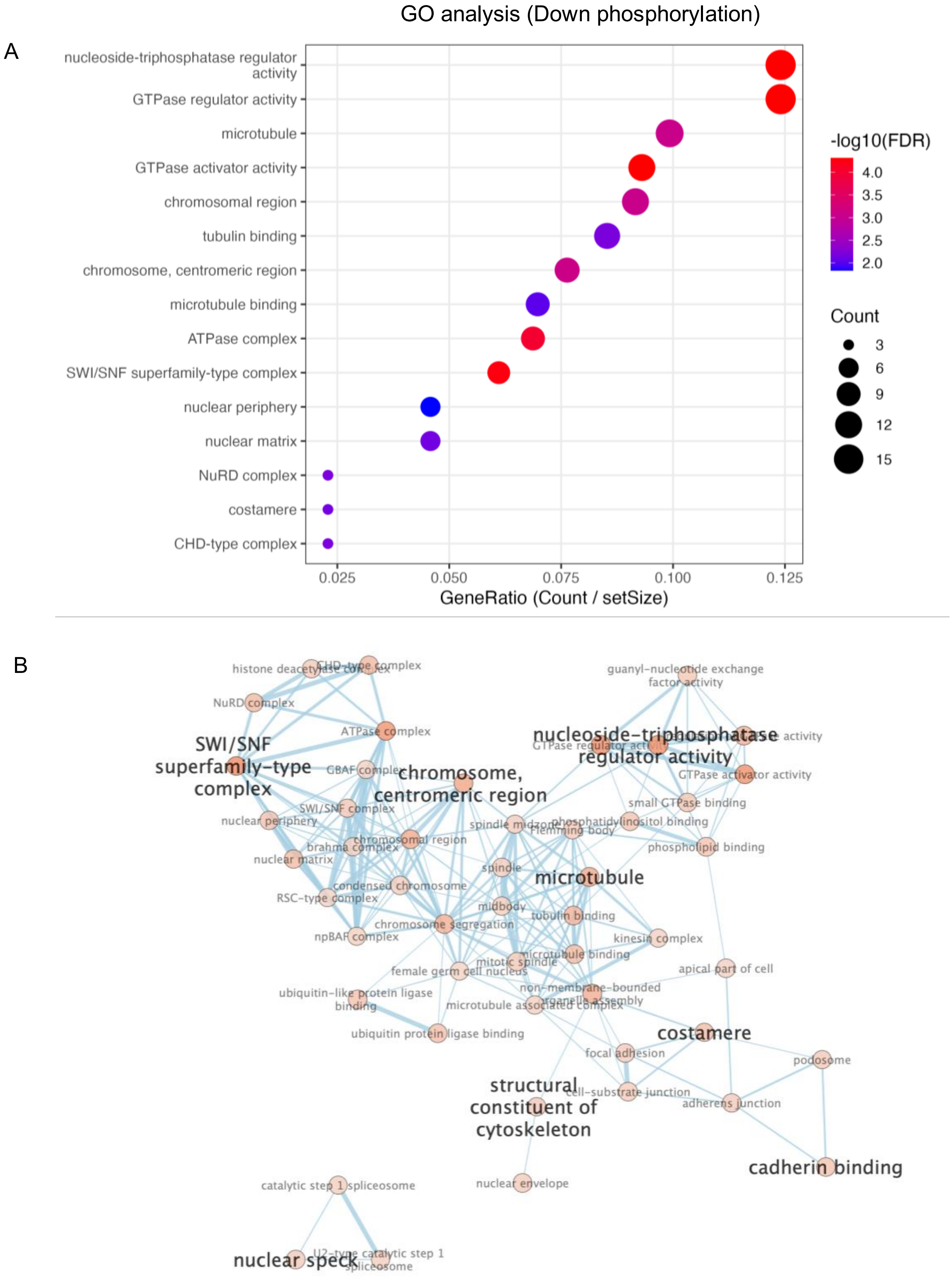

Supplementary Figure 3. GO enrichment analysis of downregulated phosphoproteins after Yoda1 stimulation.

(A) Dot plot of the top 15 enriched Gene Ontology (GO) terms for phosphoproteins whose phosphorylation decreased after Yoda1 stimulation.

(B) Network visualization of functionally related GO terms. Terms associated with microtubule/tubulin binding, GTPase regulator/activator activity, and chromosomal/centromeric region were significantly enriched. These findings indicate that phosphoproteins showing decreased phosphorylation after Yoda1 treatment were enriched in terms related to microtubule/tubulin binding, GTPase regulation, and chromosomal/centromeric regions.

Supplementary Figure 4

A

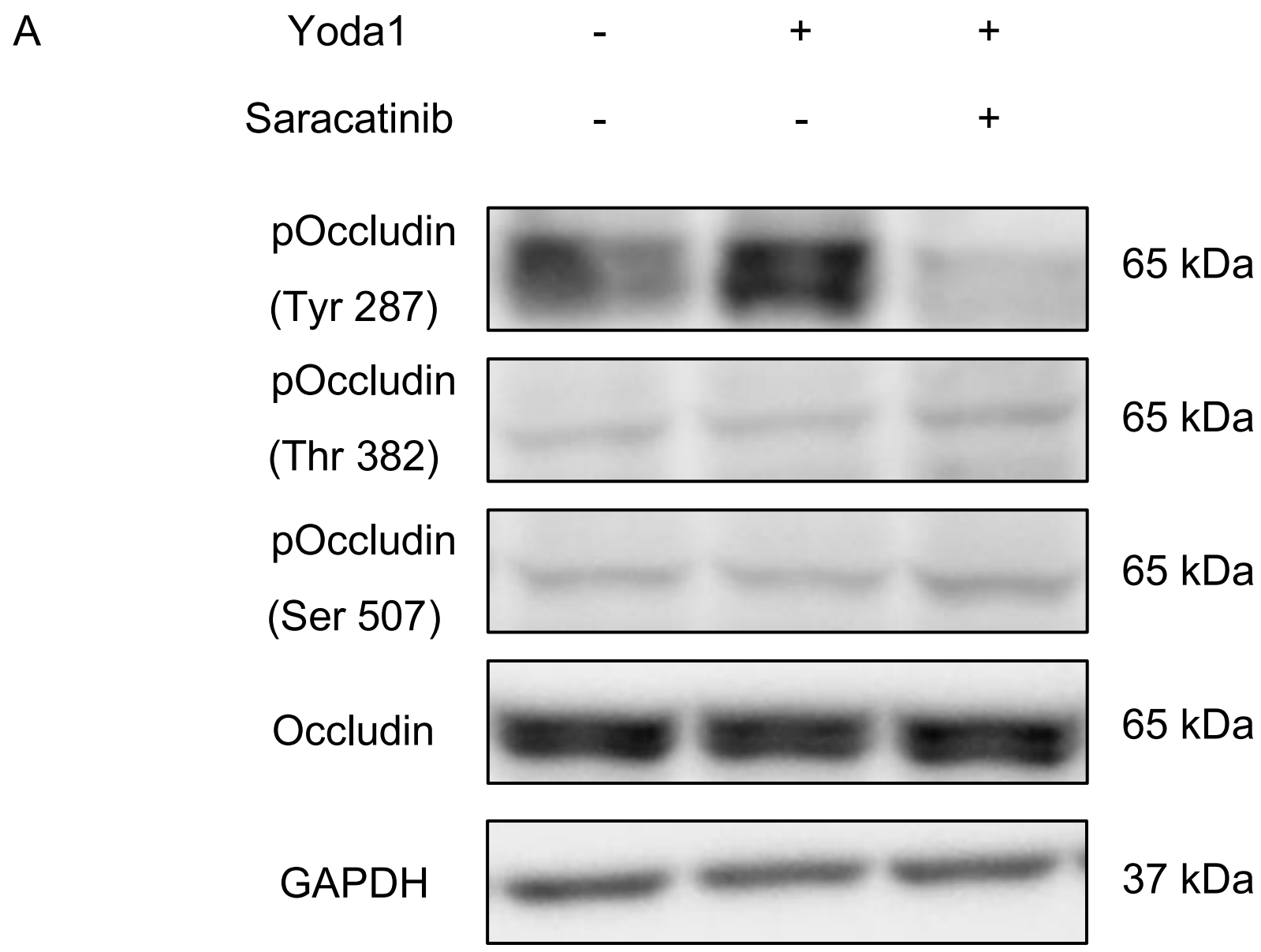

B

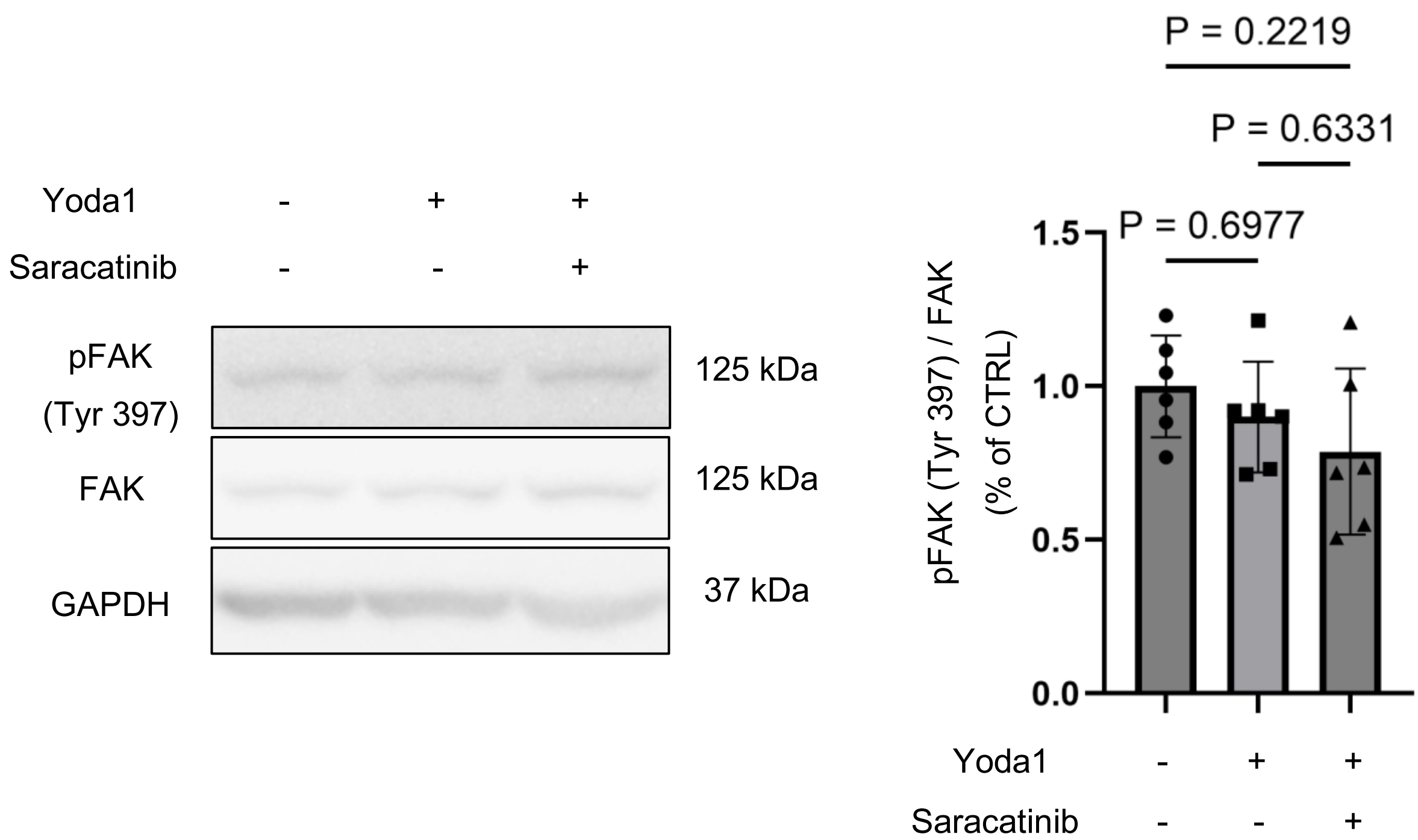

Supplementary Figure 4. Phosphorylation status of occludin and FAK after Yoda1 stimulation.

Western blot analysis showing the phosphorylation levels of occludin and FAK in HBEC-5i cells treated with Yoda1 with or without the Src inhibitor saracatinib.

(A) Phosphorylation of occludin at Tyr287 was increased by Yoda1 stimulation, whereas phosphorylation at Thr382 and Ser507 showed no marked change.

(B) Phosphorylation of FAK at Tyr397 was not significantly altered by Yoda1 or saracatinib treatment. Data are presented as mean ± SD (n = 6). Group differences were analysed by one-way ANOVA followed by Tukey's post hoc test; no statistically significant differences were detected among groups for FAK Tyr397 ($P > 0.05$ for all comparisons).

Supplementary Figure 5

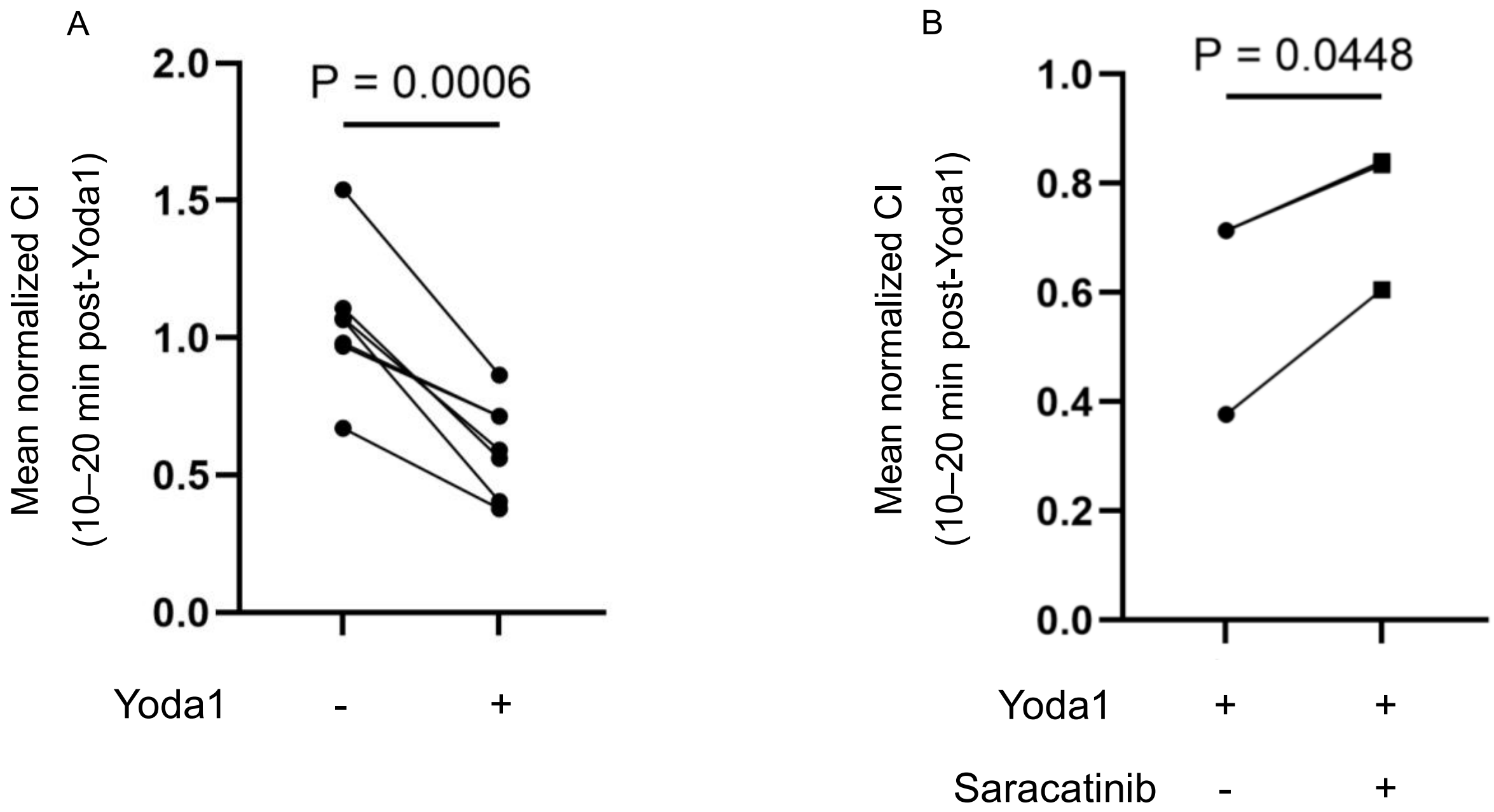

Supplementary Figure 5. Reproducibility of Yoda1-induced Cell Index reduction and its attenuation by saracatinib. For each independent experimental run, the mean normalized Cell Index during 10–20 min after Yoda1 addition was first averaged across technical-replicate wells within each condition. Each connected pair represents one independent experimental run.

(A) CTRL versus Yoda1 (15 μM), n = 7 independent experimental runs.

(B) Yoda1 (15 μM) versus Yoda1 plus saracatinib (5 μM), n = 3 independent experimental runs. Two data points overlap because the corresponding values are nearly identical.

Comparisons were performed using paired two-tailed Student's t-tests. Exact P values are shown.

Supplementary Figure 6

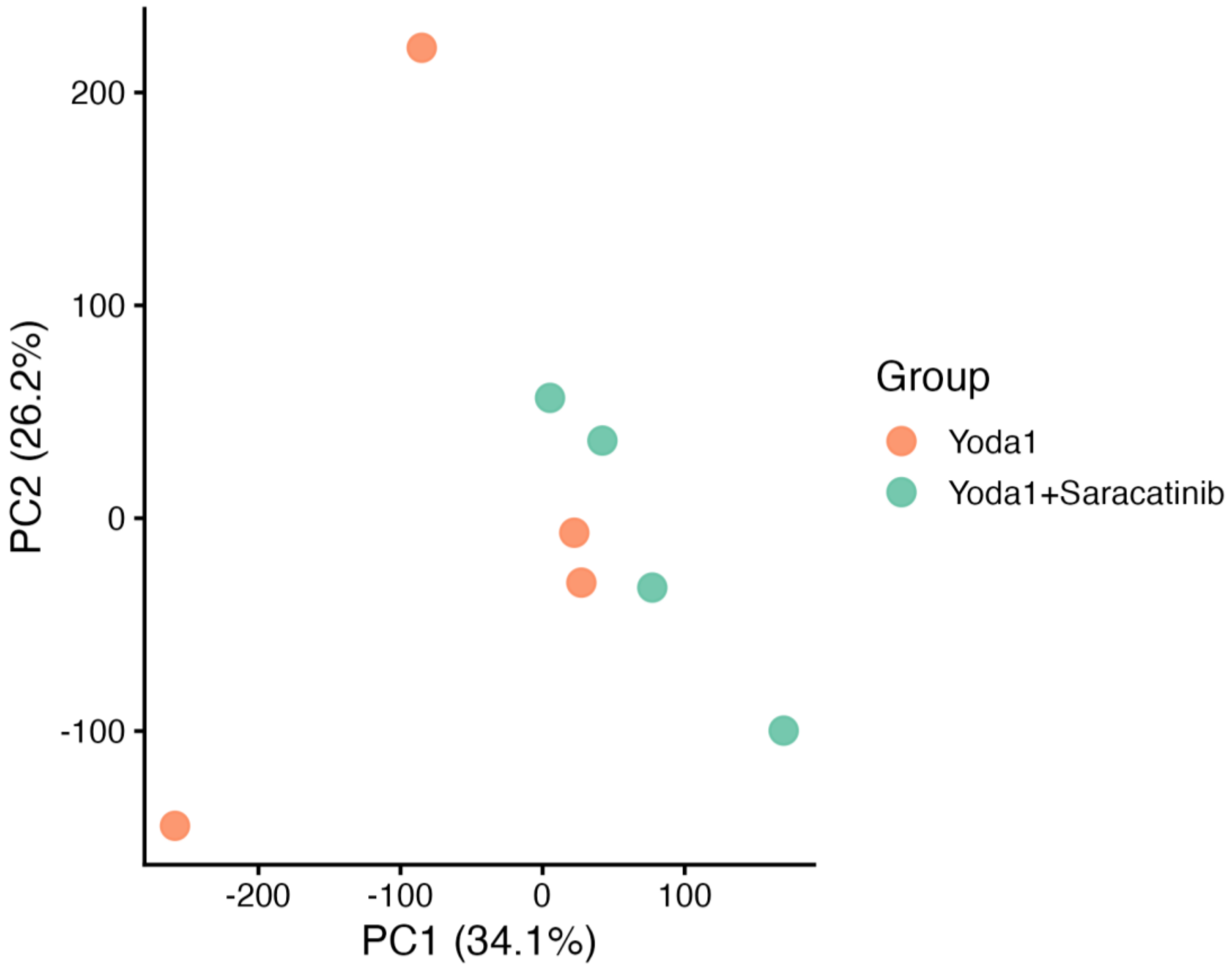

Supplementary Figure 6. Principal component analysis (PCA) of phosphoproteome profiles after Yoda1 treatment with or without saracatinib.

PCA of the phosphoproteomic dataset comparing Yoda1-treated and Yoda1 + saracatinib-treated HBEC-5i cells. No distinct separation was observed between the two groups, indicating that global phosphorylation patterns were largely similar. However, analysis of phosphopeptide-ion features meeting the exploratory criteria suggested attenuation of phosphorylation changes in the presence of saracatinib (see Fig. 4A, B).

Supplementary Figure 7

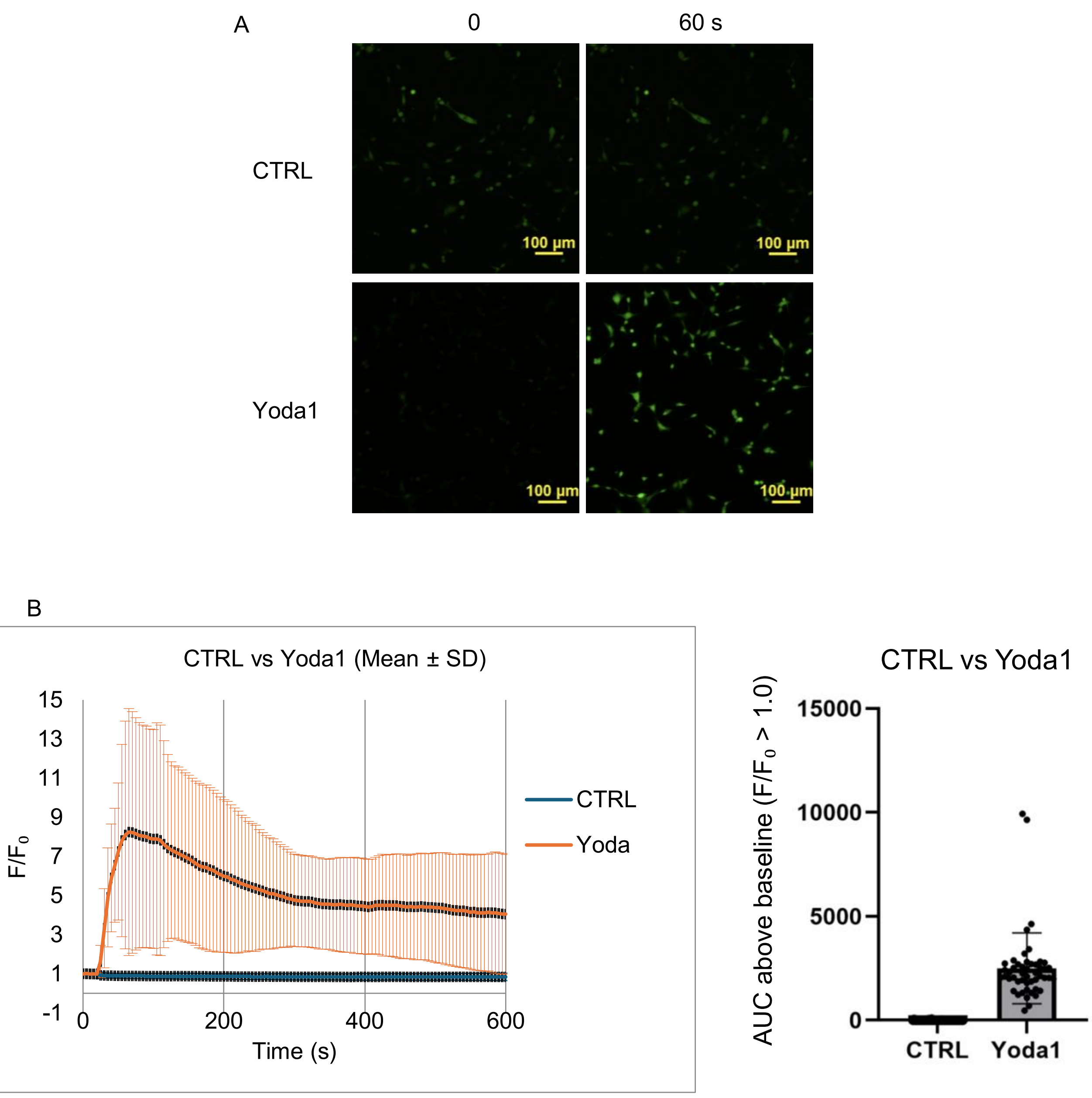

Supplementary Figure 7. Yoda1-induced intracellular Ca^{2+} influx in human brain microvascular endothelial cells (HBEC-5i).

(A) Representative live-cell fluorescence images of HBEC-5i cells stimulated with Yoda1 or vehicle. Images were acquired at baseline and 60 s after stimulation. Scale bars, 100 µm.

(B) Representative time course of intracellular Ca^{2+} responses measured using Fluo-4. Fluorescence intensity was normalized to baseline and expressed as F/F_0. The right panel shows the area under the F/F_0 curve above baseline ($F/F_0 > 1.0$) from 0 to 600 s for 50 randomly selected cells per condition within one representative experiment. Data are presented as cell-level observations and were not treated as independent biological replicates. No inferential statistical analysis was performed.

Supplementary Figure 8

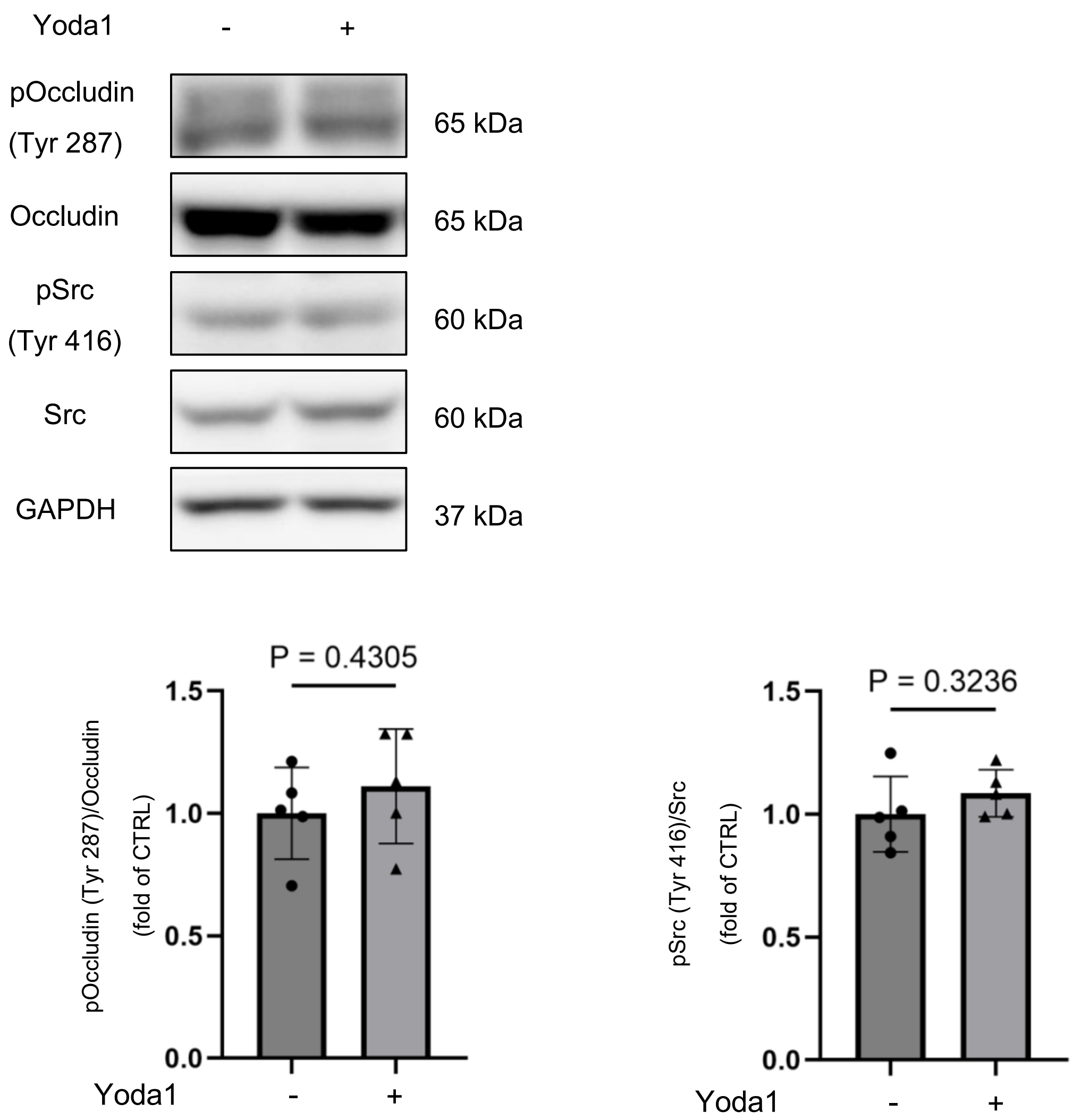

Supplementary Figure 8. Time-dependent phosphorylation of occludin and Src following Yoda1 stimulation. Western blot analysis of phosphorylated occludin (Tyr287) and phosphorylated Src (Tyr416) in HBEC-5i cells stimulated with Yoda1 (15 μM) for 5 minutes. At 1 minute after stimulation (see Figure 3A), phosphorylation levels of both pOccludin (Tyr287) and pSrc (Tyr416) were transiently increased; however, these phosphorylation changes were not observed at 5 minutes post-stimulation. Quantitative analysis of band intensities (bottom panels) confirmed that phosphorylation levels at 5 minutes were comparable to those in control cells. Data are presented as mean ± SD (n = 5). Group differences were analysed by unpaired two-tailed Student's t-test; no statistically significant differences were detected ($P > 0.05$ for both comparisons; ns). These findings indicate that Piezo1 activation induces a rapid and transient phosphorylation response in brain endothelial cells.

Supplementary Figure 9

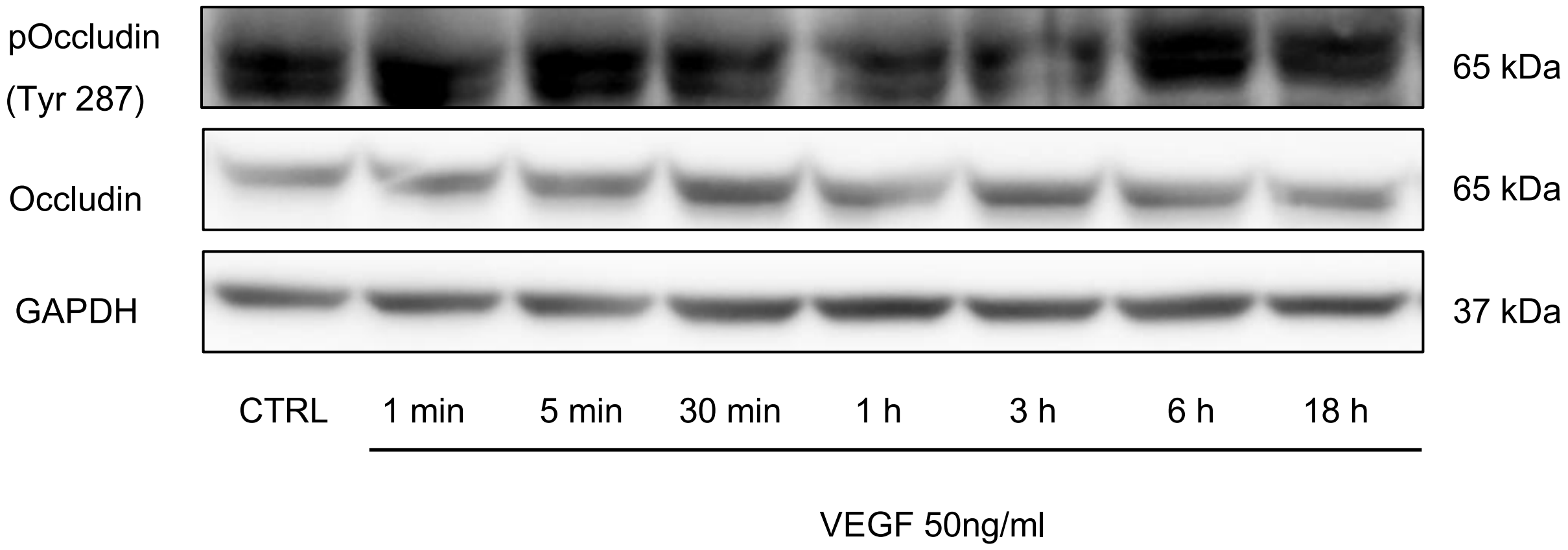

Supplementary Figure 9. Time course of occludin (Tyr287) phosphorylation after VEGF stimulation.
Western blot analysis of occludin phosphorylation at Tyr287 in HBEC-5i cells stimulated with vascular endothelial growth factor (VEGF, 50 ng/mL) for the indicated times (1 min to 18 h).
Phosphorylation at Tyr287 was not induced by VEGF at any time point examined.
VEGF did not increase occludin Tyr287 phosphorylation under the conditions examined. This finding indicates that Tyr287 phosphorylation is not invariably induced by a permeability-promoting stimulus but does not establish pathway specificity.
