# Supplementary material for: Piezo1–Src signaling rapidly disrupts brain endothelial barrier integrity
Source: Fluids Barriers CNS. 2026 Aug 13;23:112. doi: 10.1186/s12987-026-00859-6 (PMC13613767; doi:10.1186/s12987-026-00859-6)
Supplement: Supplementary file 3 [file 12987_2026_859_MOESM2_ESM.docx]

**Supplementary Methods**

**Cell proliferation assay (CCK-8)**
Cell proliferation was assessed using the CCK-8 assay (Dojindo, Kumamoto, Japan). 96-well plates were coated with gelatin by incubating at room temperature for 45 minutes, followed by removal of excess gelatin. HBEC-5i cells were seeded at a density of 5000-7000 cells/well. The CCK-8 assay was performed on the following day (approximately 24 hours after seeding). Absorbance at 450 nm was measured using a microplate reader, and relative cell viability/proliferation were calculated after subtracting the background absorbance of medium-only wells. Cells were treated with Yoda1, saracatinib, or defactinib at the indicated concentrations f**or** 24 hours before the CCK-8 assay.

**Intracellular Ca²⁺ imaging**
Fluorescence imaging of intracellular Ca^2+^ were performed as previously described. [10] Cells were incubated with Hanks’ Balanced Salt Solution (HBSS+; Wako) buffer containing 4 µM of Fluo-4 AM (Dojindo, Kumamoto, Japan), followed by washing and incubation with HEPES-buffered saline containing 2.0 mM of CaCl2. An Eclipse Ti (Nikon Corporation, Tokyo, Japan) was used to monitor fluorescence changes. Full images were collected every 5 s. Images were acquired every 5 s using excitation and emission wavelengths of 488 and 522.5 nm, respectively, and the data were expressed as background-corrected fluorescence normalized to baseline (F/F₀).

The Ca²⁺ response was quantified as the area under the F/F₀ curve above baseline (F/F₀ > 1.0) from 0 to 600 s using the trapezoidal rule (AUC above baseline). For each condition, AUC values were calculated for 50 randomly selected cells within a representative imaging field. Cell-level data were used to describe the distribution of Ca²⁺ responses within this representative experiment and were not treated as independent biological replicates.

**Live-cell imaging**
Confluent HBEC-5i monolayers in gelatin-coated 24-well plates were imaged every 5 s under CTRL, Yoda1 (15 µM), or Yoda1 (15 µM) + saracatinib (5 µM) using an Eclipse Ti microscope (Nikon) at 37 °C. Time-lapse imaging was performed for 40 min at ×160 magnification. The representative videos are provided as Supplementary Movies: CTRL (Supplementary Movie 1), Yoda1 (Supplementary Movie 2), and Yoda1 + saracatinib (Supplementary Movie 3).
